# Dual Activation of Phosphodiesterase 3 and 4 Regulates Basal Cardiac Pacemaker Function and Beyond

**DOI:** 10.3390/ijms22168414

**Published:** 2021-08-05

**Authors:** Tatiana M. Vinogradova, Edward G. Lakatta

**Affiliations:** Laboratory of Cardiovascular Science, Intramural Research Program, National Institute on Aging, National Institute of Health, 251 Bayview Boulevard, Baltimore, MD 21224, USA; lakattae@grc.nia.nih.gov

**Keywords:** sinoatrial node, phosphodiesterase, adenylyl cyclase, cardiac pacemaker cells, local Ca^2+^ release, sarcoplasmic reticulum, phospholamban

## Abstract

The sinoatrial (SA) node is the physiological pacemaker of the heart, and resting heart rate in humans is a well-known risk factor for cardiovascular disease and mortality. Consequently, the mechanisms of initiating and regulating the normal spontaneous SA node beating rate are of vital importance. Spontaneous firing of the SA node is generated within sinoatrial nodal cells (SANC), which is regulated by the coupled-clock pacemaker system. Normal spontaneous beating of SANC is driven by a high level of cAMP-mediated PKA-dependent protein phosphorylation, which rely on the balance between high basal cAMP production by adenylyl cyclases and high basal cAMP degradation by cyclic nucleotide phosphodiesterases (PDEs). This diverse class of enzymes includes 11 families and PDE3 and PDE4 families dominate in both the SA node and cardiac myocardium, degrading cAMP and, consequently, regulating basal cardiac pacemaker function and excitation-contraction coupling. In this review, we will demonstrate similarities between expression, distribution, and colocalization of various PDE subtypes in SANC and cardiac myocytes of different species, including humans, focusing on PDE3 and PDE4. Here, we will describe specific targets of the coupled-clock pacemaker system modulated by dual PDE3 + PDE4 activation and provide evidence that concurrent activation of PDE3 + PDE4, operating in a synergistic manner, regulates the basal cardiac pacemaker function and provides control over normal spontaneous beating of SANCs through (PDE3 + PDE4)-dependent modulation of local subsarcolemmal Ca^2+^ releases (LCRs).

## 1. Introduction

The sinoatrial (SA) node, the primary physiological pacemaker of the heart, is responsible for generation of every normal heartbeat, and during a human lifespan, the SA node creates more than 2.8 billion heartbeats. The SA node automaticity is generated within SA node pacemaker cells (SANCs), which fire spontaneous action potentials (APs) because of gradual depolarization of the membrane potential called diastolic depolarization (DD) [1]. Despite many achievements in the last 20 years, many mysteries about mechanisms of cardiac pacemaking and its intrinsic regulation remain unsolved. Several features separate cardiac pacemaker cells from atrial or ventricular myocytes. Morphologically, SANC are mostly spindle-shaped and much smaller (cell capacitance ~30–70 pF) compared to atrial or ventricular myocytes, and they do not have t-tubules. SANC do not have the inward rectifier potassium current I_K1_ as well as its stabilizing effect on the resting membrane potential. As a result, compared to atrial or ventricular myocytes, SANC have high membrane resistance, which allow small ion currents to produce a substantial effect on the membrane potential. Nowadays, it is well recognized that spontaneous beating of cardiac SANC is governed by a coupled-clock system (discussed below), which has overlapping and redundant systems to create robust and reliable automaticity [2,3,4,5]. Within the SA node, individual SANC continually respond to intrinsic signals generated at the subcellular, cellular, and tissue levels, as well as extrinsic signals from the autonomic nervous system and various circulating and locally released factors. 

Since its discovery, the cAMP signaling pathway has emerged as an evolutionarily highly conserved mechanism, involved in regulation of multiple physiological responses across various cell types. Phosphodiesterases (PDEs) are the only enzymes that degrade cAMP and terminate cyclic nucleotide signal, controlling cAMP levels and spatial distribution within the cell. Both PDE3 and PDE4 preferentially hydrolyze cAMP and are among the most widely distributed and abundant PDE isoforms [6]. Regulation of cAMP by synergistic dual PDE3 and PDE4 activation has been recognized in many different cell types, as diverse as cardiac myocytes, SANC, brown adipose tissue, and smooth muscle cells [7,8,9,10,11], suggesting conservation of dual PDE3 and PDE4 function among various cell types of multiple species. Currently, dual PDE3 + PDE4 inhibitors are accepted to treat patients with allergic rhinitis, asthma, and chronic obstructive pulmonary disease (COPD). In clinical trials, dual PDE3/PDE4 inhibitor (RPL554: Verona Pharma) improved lung function without undesirable side effects of “classical” PDE4 inhibitors [12,13]. Synergistic effects of dual PDE3 + PDE4 inhibition markedly increases drug efficacy, improving conditions of patients with asthma and COPD [13,14].

The aim of this review is to highlight one of the essential intrinsic mechanisms, i.e., the role of dual PDE3 and PDE4 activation in the regulation of the basal cardiac pacemaker function. Here, we first provide a brief overview of the “coupled-clock” pacemaker system that drive SA node automaticity, to introduce the primary targets for modulation of the SA node beating rate by basal PDE activation. This is followed by description of high basal level of cAMP in cardiac pacemaker cells created by constitutive adenylyl cyclase (AC) activation and consequent augmentation of phosphorylation by protein kinase A (PKA) and Ca^2+^/calmodulin-dependent protein kinase II (CaMKII) of proteins involved in the generation of SA node automaticity. Then we introduce PDEs, which constantly degrade cAMP to keep cAMP and cAMP-mediated PKA-dependent phosphorylation under control, and synergistic effect of dual PDE3 + PDE4 activation on PKA-dependent protein phosphorylation and spontaneous beating rate of SANC. Finally, we will describe how dual PDE3 + PDE4 activation, working in a synergistic manner, modulates specific targets of the “coupled-clock” system and the critical role of LCRs in PDE-dependent regulation of the basal cardiac pacemaker function. We will also discuss evidence for compartmentalization of cAMP signaling in cardiac pacemaker cells under basal conditions.

Though our review will be focused on the regulation of basal cardiac pacemaker function by dual PDE3 + PDE4 activation, we will also discuss how dual PDE3 + PDE4 activation regulates Ca^2+^ cycling in cells from the atria and ventricle, which benefited from more extensive experimental studies, including results obtained from the transgenic mouse models as well as human studies. It should be mentioned that there are important differences between cell types in various regions of the heart (e.g., SANC vs. atrial or ventricular myocytes) based on their diverse cardiac function, which can lead to variations in the PDE-dependent modulation of ion channels and SR Ca^2+^ cycling. 

## 2. Coupled-Clock System Regulates Spontaneous Firing of Cardiac Pacemaker Cells

**Figure 1:**
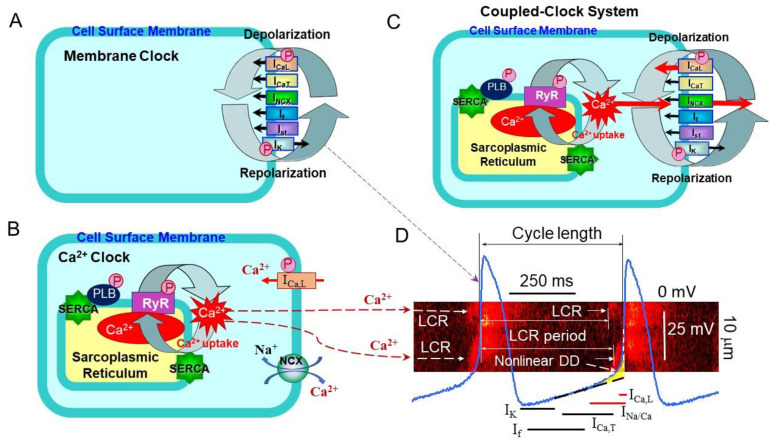
A schematic illustration of the coupled-clock pacemaker system. (**A**) Schematic presentation of ion channels “membrane clock” (including the hyperpolarization activated “funny” current I_f_, L-type and T-type Ca^2+^ currents (I_Ca,L_, I_Ca,T_), delayed rectifier potassium current (I_K_), Na^+^/Ca^2+^ exchange current (I_NCX_), Na^+^/K^+^ exchange current (I_NaK_), sustained current I_st_, etc.) and (**B**) “Ca^2+^ clock” in cardiac pacemaker cells. Note that both L-type Ca^2+^ channels and Na^+^-Ca^2+^ exchanger are part of the “membrane clock” and “Ca^2+^ clock”. Panel (**C**) illustrates the “coupled-clock” system with complex interactions between the “membrane clock” and “Ca^2+^ clock” (see text for details). (**D**) Schematic illustration of spontaneous SANC action potentials, Ca^2+^ transients, LCRs, and several major ion currents involved in generation of the diastolic depolarization (DD). LCR-induced increase in local [Ca^2+^] beneath sarcolemma activates an inward NCX current creating exponential increase in the DD rate (nonlinear DD). The LCR period represents the essence of the “coupled-clock” system, which comprises complex interactions between cell membrane electrogenic molecules and intracellular SR Ca^2+^ cycling (see text for details).

The generation of repetitive, rhythmic action potentials is the main responsibility of the sinoatrial node, specifically sinoatrial node cells (SANC). The spontaneous DD is the essence of cardiac pacemaker cell automaticity; it produces a slow spontaneous increase in the membrane potential toward an excitation threshold to fire an action potential. Initially, it was assumed that surface membrane ion channels were sufficient at generating spontaneous DD and spontaneous beating of cardiac pacemaker cells. The cardiac pacemaker field adopted the idea of DD based on voltage- and time-dependent surface membrane channels, envisioned as the “membrane clock”, which regulates normal automaticity of cardiac pacemaker cells (Figure 1A). Several voltage-gated ion channels and transporters in the cell membrane have important contribution in the ‘membrane clock’, including the hyperpolarization activated “funny” current I_f_, L-type and T-type Ca^2+^ currents (I_Ca,L_, I_Ca,T_), delayed rectifier potassium current (I_K_), Na^+^/Ca^2+^ exchange current (I_NCX_), Na^+^/K^+^ exchange current (I_NaK_), etc. (only the most important currents are shown in Figure 1A) [1,4,15,16,17].

Similar to other cardiac cells, SANCs have the sarcoplasmic reticulum (SR) and are equipped to cycle Ca^2+^ via SR Ca^2+^-ATPase (SERCA2) and Ca^2+^ release channels, ryanodine receptors (RyR). Confocal microscopy and Ca^2+^-sensitive fluorescent probes allowed to identify spontaneous, roughly periodic, local Ca^2+^ releases (LCRs) during late DD beneath the sarcolemma of cardiac pacemaker cells [18] (Figure 1B). Further studies confirmed the presence of rhythmic spontaneous LCRs under normal physiological conditions in SANC of different species [19,20,21,22,23].

During each spontaneous cycle, Ca^2+^ influx through L-type Ca^2+^ channels, triggered by the AP upstroke, produces a global Ca^2+^ transient, emptying of the SR Ca^2+^ store and averting LCR generation. When the SR Ca^2+^ content is refilled by SERCA, which constantly pumps Ca^2+^ back into the SR, LCRs begin to occur, and the cycle begins once more. The restitution time, the time from the AP triggered global Ca^2+^ transient to the onset of LCRs during DD is the LCR period (Figure 1D). LCRs do not require change in the membrane potential and continue to occur during voltage clamp of the cell membrane or in saponin-permeabilized SANCs [2,20,24], manifesting intracellular SR Ca^2+^ cycling of “Ca^2+^ clock” in the absence of the “membrane clock” (Figure 1B). Saponin treatment partially removes cell sarcolemma and makes it permeable to small ions and molecules without disrupting SR function. When the cell membrane is permeabilized to remove membrane currents, Ca^2+^ cycling by the SR becomes “free running” and is controlled mostly by the concentration of free cytosolic Ca^2+^ and the kinetics of Ca^2+^ pumping into and releasing from the SR. At the same physiological Ca^2+^ concentration, permeabilized SANC cycled Ca^2+^ beneath the sarcolemma more efficiently compared to permeabilized ventricular myocytes. Specifically, permeabilized SANC generated larger and more rhythmic spontaneous SR Ca^2+^ releases than ventricular myocytes at the similar SR Ca^2+^ content in both cell types [24].

To generate an AP, the “membrane clock” interacts with the “Ca^2+^-clock” via multiple Ca^2+^ and voltage-dependent mechanisms creating a “coupled-clock” system (Figure 1C). There are many points where the function of the two clocks overlaps, e.g., both L-type Ca^2+^ channels and the Na^+^/Ca^2+^ exchanger have dual affiliation as members of both “membrane clock” and “Ca^2+^ clock”. In intact SANC L-type Ca^2+^ channels provide Ca^2+^ supply to pump into SR, while the LCR occurrence beneath sarcolemma activates an inward Na^+^-Ca^2+^ exchange current (I_NCX_), which produces an exponential increase of the late DD rate (nonlinear DD) prompting the “membrane clock” to generate the next AP upstroke and, thus, modulating the spontaneous SANC beating rate (Figure 1B). Colocalization of Na^+^/Ca^2+^ exchanger and RyRs beneath the sarcolemma of rabbit SANC [25] permits a quick conversion of LCRs into changes in the inward I_NCX_ current that depolarizes the membrane potential. Though the contribution of the L-type Ca^2+^ channels and Na^+^-Ca^2+^ exchanger for intracellular [Ca^2+^]_i_ balance and the LCR period in SANC seems obvious, other channels, e.g., potassium channels, could also indirectly participate in adjusting the intracellular [Ca^2+^]_i_ balance. Indeed, potassium channels repolarize the membrane potential and, therefore, inactivate L-type Ca^2+^ channels indirectly affecting [Ca^2+^]_i_ balance and the LCR period. The coupled-clock system function together on a beat-to-beat basis and comprises complex crosstalk between the two clocks via signaling pathways, which can modulate each other to safeguard robustness and reliability of function (Figure 1C,D) [2,3,4,5]. A perturbation of one clock inevitably affects the other due to subsequent indirect effects, resulting in mutual entrainment, e.g., inhibition of the I_f_ current by ivabradine also slows spontaneous SANC firing, leading to a decrease in the SR Ca^2+^ load and suppression of LCRs [26].

The coupled-clock system is regulated not only by Ca^2+^ or voltage-dependent mechanisms, but also by phosphorylation status of multiple proteins, which comprise both “membrane clock” and “Ca^2+^ clock” (Figure 1) (discussed below). Important phosphorylation sites exist on phospholamban (PLB), which regulates activity of SERCA, L-type Ca^2+^ channels (modulating I_CaL_), potassium channels (modulating I_K_), and RyR (likely increasing its calcium sensitivity [27]). Phosphorylation acts on both clocks and results of model simulations demonstrated that changes in the phosphorylation status are linked to changes in the degree of synchronization of the coupled-clock system [28].

## 3. Ca^2+^-Activated ACs, cAMP Synthesis, and Its Relevance for the Spontaneous Beating Rate of SANC

cAMP is a universal second messenger that coordinates a multitude of downstream intracellular signaling, and synthesis of cAMP from adenosine triphosphate (ATP) is regulated by the enzyme adenylyl cyclase (AC). The AC family consists of nine membrane-bound isoforms (AC1–9) and one soluble isoform [29]. All AC isoforms, except AC8, could be found in adult ventricular myocytes with AC5 and AC6 as major AC isoforms with lower levels of AC2, AC4, and AC9 [30]. Though AC5 and AC6 are closely related isoforms, they seem to play distinct roles in the regulation of cardiac function [30,31]. AC5 has been shown to be the dominant isoform in the heart and has the highest enzyme catalytic activity among AC isoforms [32]. The most notable effect of AC5 deletion is elimination of parasympathetic control of cAMP levels in AC5-KO mice, as well as protection of the heart against chronic β-AR stimulation, suggesting that the β1-adrenergic receptor selectively couples to AC5 [33,34]. Deletion of AC6 was associated with reduced left ventricular contractile function, decreased PKA activity, and marked abnormalities in Ca^2+^ transients of cardiac myocytes due to decreased PLB phosphorylation and impaired activity of SR Ca^2+^-ATPase [35]. In cardiomyocytes, cAMP is produced by ACs generally in response to stimulation of catecholamine- or hormone-activated receptors; cAMP has several major downstream targets, including protein kinase A (PKA), exchange protein activated by cAMP (EPAC), nucleotide gated ion channels, and PDEs, which degrade cAMP into 5′-AMP terminating its ability to modulate downstream targets.

Though all ACs are inhibited by high intracellular Ca^2+^ [Ca^2+^]_i_ [36], AC5 and AC6 are inhibited at the physiological range (0.1–1µmol/L) of [Ca^2+^]_i_. In contrast, AC1 and AC8 are stimulated by [Ca^2+^]_i_ in a CaM-dependent manner, and AC 2/4/7/9 are Ca^2+^ insensitive. Different AC isoforms localize to distinct membrane compartments: Ca^2+^-sensitive ACs (AC1/3/5/6/8) were found in lipid rafts structures, while Ca^2+^ insensitive ACs (AC 2/4/7/9) were excluded [29]. Therefore, lipid rafts and by extension caveolae, likely represent specific cellular regions that can promote and strengthen interactions between multiple signaling targets [29].

The idea that intracellular cAMP is required for cardiac pacemaker function was first proposed more than four decades ago and based on the observation that exposure to AC inhibitors suppressed spontaneous beating of isolated rabbit SA node [37], and this effect was reversed by dibutyryl cAMP [38]. Though inhibition of ACs in this original study was indirect, further experiments using an iontophoretic injection of cAMP into Purkinje fibers, or superfusion of isolated rabbit SA node with a dibutyryl cAMP containing solution, produced a marked increase in the DD slope, and a concomitant increase in the spontaneous beating rate [38,39], confirming the link between changes in intracellular cAMP and spontaneous firing of cardiac pacemaker tissue. 

**Figure 2:**
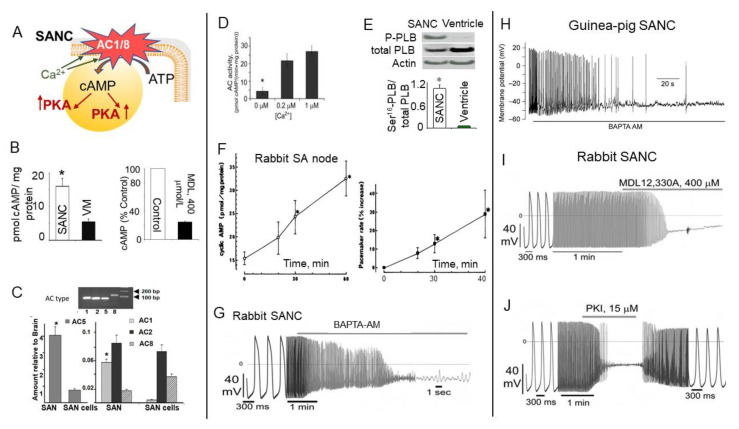
Constitutive AC activation, high basal level of cAMP, and PKA-dependent protein phosphorylation is central for spontaneous firing of cardiac pacemaker cells. (**A**) Cartoon representing constitutive activation of ACs, high basal level of cAMP, and PKA-dependent phosphorylation in SANC. (**B**) Left, average basal cAMP in SANCs or ventricular myocytes (modified from [41]); right, AC inhibitor MDL-12,330A markedly decreases cAMP in SANC (modified from [40]). (**C**) Abundance of AC isoform transcripts in the rabbit SA node or SANC relative to brain tissues (from [42]). (**D**) Presence of Ca^2+^-stimulated AC activity in whole cell lysates of intact rabbit SANC (from [42]). (**E**) Basal level of PLB phosphorylation at PKA-dependent Ser^16^ site is significantly higher in SANC compared to ventricular myocytes (VM) (modified from [40]). (**F**) Time course of the effect of AC activation by cholera enterotoxin on cyclic AMP content (left) and pacemaker rate (right) of the rabbit SA node preparation (with permission, from [43]). (**G**–**J**) Ca^2+^ chelation, AC or PKA inhibition suppresses spontaneous SANC beating rate; representative examples of: (**G**) effects of Ca^2+^ chelation by 25 µmol/L BAPTA-AM in rabbit SANC (from [42]) and (H) 10 µmol/L BAPTA-AM in guinea pig SANC (with permission from [44]); (**I**) effects of AC inhibition by MDL-12,330A (400 µmol/L) and (**J**) PKA inhibition by specific PKA inhibitory peptide PKI (15 µmol/L) (**I**–**J** from [42]).

Direct measurements of cAMP in isolated rabbit SANC were made three decades later and revealed that basal level of cAMP in intact isolated SANC is ~3-fold higher compared to ventricular myocytes (Figure 2A,B (left)). This high basal level of cAMP (in the absence of any membrane receptor agonists) in SANC is due to constitutive activation of ACs and is markedly suppressed by the AC inhibitor, MDL-12,330A (Figure 2B (right)) [40,41]. Constitutive AC activation could not be explained by constitutive activation of beta-adrenergic receptors (β-AR), because either β1-AR antagonist, CGP-20712A, or the β2-AR subtype inverse agonist, ICI 118,551, failed to alter the normal spontaneous SANC firing rate [2,40,42].

An examination of this ongoing basal activity of ACs demonstrated association between Ca^2+^ and cAMP indicating a possible link to Ca^2+^-activated type of ACs. SANCs, like ventricular myocytes, express AC5/6 but they also express Ca^2+^-activated AC1 and AC8: AC1 was reported in the guinea pig SA node [45], and both AC1 and AC8 were found in rabbit SA node and SANC [42] (Figure 2C). The latter study also showed that Ca^2+^-activated AC isoforms localize to membrane lipid microdomains and are activated by Ca^2+^ (Figure 2D), increasing cAMP production over the wide physiologic range (0.2–1 µmol/L) of intracellular [Ca^2+^]_i_ [42]. This association between Ca^2+^ and cAMP was further confirmed in permeabilized SANC (in the absence of functional ion channels), i.e., an increase in cytosolic [Ca^2+^]_i_ was accompanied by an increase in the cAMP level, indicating activation of ACs and consequently an increase in the cAMP production [24]. Transgenic mice overexpressing AC8 have increased level of cAMP and a faster heart beating rate [46,47].

PKA is the main downstream target of cAMP. Since mechanisms of PLB phosphorylation via PKA are similar to mechanisms responsible for phosphorylation of other major proteins regulating SANC automaticity, the phosphorylation status of PLB could be used as an index of global phosphorylation status of SANC [2,40]. Intact SANC have high basal level of PLB phosphorylation at PKA-dependent Ser^16^ site, which by ~10-fold exceeds that in ventricular myocytes (Figure 2E), indicating high basal level of cAMP-mediated PKA-dependent phosphorylation of multiple proteins in cardiac pacemaker cells. Inhibition of ACs not only reduced the level of cAMP (Figure 2B, right), but also suppressed PKA-dependent protein phosphorylation, confirming a direct link between AC activation and basal protein phosphorylation in cardiac pacemaker cells [40]. Although an increase in cytosolic [Ca^2+^]_i_ had no effect on PKA-dependent PLB phosphorylation in permeabilized ventricular myocytes, it markedly increased PLB phosphorylation in permeabilized SANC, indicating that high basal PKA-dependent protein phosphorylation in SANC is due to constitutive activation of Ca^2+^-activated ACs [24].

Maneuvers known to regulate basal cAMP-mediated, PKA-dependent signaling also affect the normal SA node spontaneous beating rate. Indeed, stimulation of AC by cholera enterotoxin time-dependently increased level of cAMP, which was paralleled by an acceleration of spontaneous SA node beating rate [43] (Figure 2F). The dependence of spontaneous SANC firing rate on intracellular [Ca^2+^]_i_ was further confirmed by the observation that spontaneous activity of single SANC isolated from either rabbit or guinea pig SA node is abrogated by chelation of cytosolic Ca^2+^ with intracellular BAPTA (Figure 2G and 2H, respectively). This effect of Ca^2+^ buffering happened, in part at least, via a reduced ability of Ca^2+^ to activate AC1/8, leading to a local reduction of cAMP and PKA-dependent protein phosphorylation. 

A key role of constitutive AC activation and PKA-dependent phosphorylation for normal spontaneous beating of single isolated SANC was demonstrated using selective AC inhibitor MDL-12,330A, which reduced the level of cAMP (Figure 2B, right) and concurrently decreased and finally abrogated spontaneous SANC firing (Figure 2I) [42]. This effect was partly due to the decrease in the PKA-dependent protein phosphorylation, since the membrane-permeable PKA inhibitor peptide (PKI) slowed and abolished spontaneous SANC beating; this effect was reversible on PKI washout (Figure 2J) [42]. Finally, numerical model simulations supported hypothesis that there is a direct link between changes in Ca^2+^-activated AC-cAMP-PKA signaling and spontaneous beating rate of cardiac pacemaker cells [48].

## 4. Phosphorylation of “Membrane Clock” and “Ca^2+^ Clock” Proteins in SANC Safeguards Function of the Coupled-Clock System

Basal AC activity and high level of cAMP increases PKA-dependent protein phosphorylation in SANC (Figure 2) amplifies Ca^2+^ influx through L-type Ca^2+^ channels [49] and AP-induced Ca^2+^ transients, increasing local and global Ca^2+^ concentrations. Signals that increase [Ca^2+^]_i_ activate CaMKII [50]. Active (autophosphorylated) CaMKII is located in microdomains of the surface membrane of SANC [51] in close proximity to L-type Ca^2+^ channels and RyR [25,51], and it can retain its activity even in the absence of further increase in [Ca^2+^]_i_ [52]. Based on its “memory” properties [53] CaMKII could serve as a “frequency detector” and integrate local Ca^2+^ signals, reflecting performance of SANC: the faster SANC beats and more frequent are local Ca^2+^ releases, the greater is the CaMKII activity. The basal level of activated (autophosphorylated) CaMKII in rabbit SANC by ~2-fold exceeds that in ventricular myocytes [54]. Close connection between PKA- and CaMKII-dependent phosphorylation has been recently confirmed in mice expressing a PKA inhibitor peptide in cardiomyocytes (cPKAi) with almost complete inhibition of cardiac PKA activity [55]. The reduction of PKA activity led to a markedly decreased level of CaMKII phosphorylation in cardiomyocytes as well as CaMKII-dependent phosphorylation of PLB and RyR2 [55].

The high basal protein phosphorylation by both PKA and CaMKII is required for normal coupled-clock pacemaker function because spontaneous AP firing ceases when either PKA- or CaMKII-dependent phosphorylation is inhibited [40,51,54,56]. Both PKA and CaMKII share the same phosphorylation targets, including L-type Ca^2+^ channels, PLB and RyR2 (Figure 1). L-type Ca^2+^ channels in SANC are part of both “membrane clock” and “Ca^2+^ clock”, since they generate action potential upstroke and at the same time provide Ca^2+^ supply for pumping into SR. L-type Ca^2+^ channels in SANC are highly phosphorylated by both PKA and CaMKII in the basal state, as specific PKA inhibitor peptide, PKI, or CaMKII inhibitors, KN-93 or AIP, suppressed I_CaL_ by ~80% [49] and ~50% [51], respectively. Delayed rectifier potassium channels are also phosphorylated by PKA, i.e., stimulation of PKA with the membrane-permeable cAMP analog by ~2-fold increased the amplitude of the delayed-rectifier potassium current (I_K_) in guinea pig ventricular myocytes [57]. In rabbit SANC, β-AR stimulation increased I_K_ current amplitude by ~70%, markedly shortened the decay of I_K_, and all effects were reversed by PKA inhibitor H-89 [58], indicating that I_K_ is a target of PKA-dependent phosphorylation (Figure 1). 

Similar to ventricular myocytes, the SR “Ca^2+^ clock” in rabbit SANC is wired to cycle Ca^2+^, and it is tightly regulated by both PKA- and CaMKII-dependent protein phosphorylation in the basal state. The SR Ca^2+^-ATPase (SERCA) pumps Ca^2+^, entering through the L-type Ca^2+^ channels, back into SR to refill the SR Ca^2+^ content and prepare for the next spontaneous cycle. Activation of SERCA accelerates re-uptake of Ca^2+^ into SR, shortens duration of AP-induced Ca^2+^ transients, and reduces duration of Ca^2+^ sparks [59]. Transgenic mice overexpressing SERCA2a protein showed increased SR Ca^2+^ uptake function, i.e., an increase in SERCA2a protein levels by ~1.5-fold in transgenic mice was associated with acceleration of the maximum velocity of SR Ca^2+^ uptake by ~40% [60]. Moreover, hearts from mice overexpressing SERCA2a showed significantly higher myocardial contractility and slightly increased spontaneous beating rate [61], signifying SERCA as a key determinant of both cardiac contraction and spontaneous SANC firing. 

Similar to atrial myocytes [62], an expression of SERCA protein in rabbit SANC is ~1.5-fold higher compared to ventricular myocytes [54]. In line with increased amount of SERCA protein, the total LCR signal mass released by permeabilized SANC (in the absence of functional ion channels) at physiological cytosolic [Ca^2+^] (150–250 nmol/L) was ~2-fold larger compared to permeabilized ventricular myocytes despite similar SR Ca^2+^ content in both cell types [24,56]. Moreover, the elevated Ca^2+^ release from the SR produced no detectable depletion of the SR Ca^2+^ content indicating more efficient SR Ca^2+^ pumping in SANC [24]. 

PLB is a functional “brake” on SERCA, and abundance of PLB protein in many species, including humans, rabbits, guinea pigs, mice, and rats is ~2–3-fold less in atria compared to ventricle [62,63]. Abundance of PLB protein in rabbit SANC is ~2-fold less than in ventricular myocytes, indicating that inhibition of SERCA by PLB in cardiac pacemaker cells is lower compared to ventricular myocytes. Considering that the amount of SERCA in SANC is ~1.5-fold higher than in ventricular myocytes, the SERCA/PLB ratio could be at least ~3-fold larger in SANC than in ventricular myocytes, which should result in increased Ca^2+^ pumping into the SR required to support robust SR Ca^2+^ release [24]. Phosphorylation of PLB by PKA or CaMKII (at Ser^16^ or Thr^17^ sites, respectively) disengage an inhibitory action of PLB on SERCA and release SERCA inhibition, elevating SERCA activity by ~2–3-fold in ventricular myocytes [64,65]. In rabbit SANC, basal PLB phosphorylation at Ser^16^ and Thr^17^ sites was respectively ~10-fold (Figure 2D) and ~3-fold [54] greater compared to ventricular myocytes, signifying high level of protein phosphorylation by both PKA and CaMKII in the basal state.

During every heartbeat, a small influx through L-type Ca^2+^ channels activate Ca^2+^-induced Ca^2+^ release (CICR) mechanism to generate Ca^2+^ release from RyR, SR Ca^2+^ release channels, amplifying the inward Ca^2+^ signal by ~10–20-fold [66,67]. The cardiac RyR2 is a large macromolecular complex connected to PKA, CaMKII, phosphatases, and PDE4D, which are hitched to the channel by A-kinase-anchoring proteins (AKAPs) [68,69,70,71]. RyR, can be phosphorylated by PKA at Ser^2030^, by CaMKII at Ser^2814/2815^ and by both PKA and CaMKII at Ser^2809^. In isolated ventricular myocytes, activation of CaMKII was associated with increased Ca^2+^ spark frequency [72]. CaMKII dependent phosphorylation substantially modifies RyR function in cardiomyocytes, increasing Ca^2+^ sensitivity of RyR and enhancing Ca^2+^ release [71]. Transgenic mice overexpressing CaMKII^d^ isoform showed elevated Ca^2+^ spark frequencies (despite lower SR Ca^2+^ content), pronounced SR Ca^2+^ leak and a susceptibility for arrhythmias linked to altered phosphorylation levels of proteins involved in Ca^2+^ handling [71,73].

Basal RyR phosphorylation at CaMKII-dependent Ser^2815^ site in rabbit SA node is ~10-fold higher compared to ventricle [54], which could be partly due to similar distribution and likely association of activated autophosphorylated CaMKII and RyR beneath sarcolemma of rabbit SANC [25,51]. Basal RyR phosphorylation at Ser^2809^ site, which is phosphorylated by both PKA and CaMKII, is also ~2-fold higher in rabbit SANC compared to ventricular myocytes [54]. Robust, rhythmic LCRs in SANC require high basal PKA- and CaMKII-dependent protein phosphorylation since inhibition of either PKA- or CaMKII-dependent phosphorylation results in small, stochastic Ca^2+^ events that resembles Ca^2+^ sparks in ventricular myocytes [24]. The coordinated and synchronized phosphorylation-driven increases in both Ca^2+^ release through RyRs and reuptake by the SR could sustain large and rhythmic spontaneous LCRs in SANC.

## 5. Requirement of PDE Activation in Cardiac Pacemaker Cells in the Basal State

**Figure 3:**
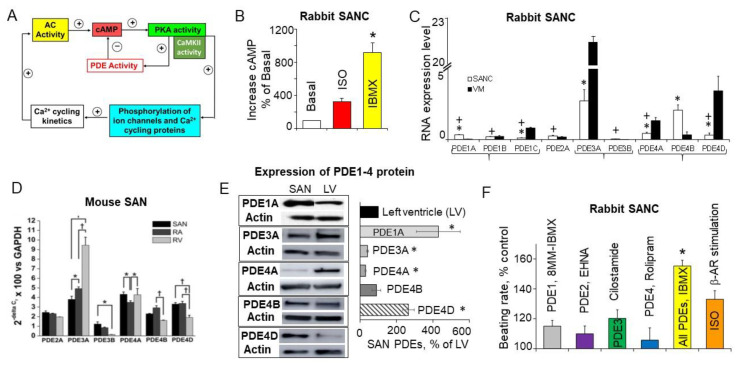
Constitutive basal PDE activation, PDE isoforms, and their role in the regulation of normal cardiac pacemaker function. (**A**) Cartoon of positive basal Ca^2+^/cAMP-PKA “feed-forward” system kept in check by high basal PDE activity, which acts as a negative feedback mechanism restricting cAMP/PKA signaling and preventing an excessive basal beating rate. (**B**) Suppression of PDE activity in SANCs markedly increases the level of cAMP exceeding effect produced by saturating concentration of β-AR agonist isoproterenol (ISO). One-way ANOVA with Bonferroni post hoc test * *p* <0.01 (IBMX vs. Basal or ISO) (modified from [41]). (**C**) Relative expression of PDE-coding transcripts (mean ± SEM) in rabbit SANCs and ventricular myocytes (VM); (n = 4–9). One-way ANOVA with Tukey post hoc test, adjusted * *p* < 0.05 (SANC vs. VM for each PDE subtype); +*p* < 0.05 (PDE subtypes in SANC vs. PDE3A or PDE4B) (from [11]). (**D**) Quantitative mRNA expression of PDE 2, 3, and 4 subtypes in mouse SA node, right atrium (RA), and right ventricle (RV). Expression of PDE2A, PDE3A, PDE3B, PDE4A, PDE4B, and PDE4D are shown relative to GAPDH as (mean ± SEM); n = 5 SA node trials, 5 right atrium (RA) trials, and 3 right ventricle (RV) trials; * *p* < 0.05; +*p* < 0.001 by two-way ANOVA with Tukey’s post-hoc test (from [88]). (**E**) (left) Representative western blots of major PDE subtypes in the rabbit SA node and left ventricle, (right) average data (n = 8) of PDE1A, PDE3A, PDE4A, PDE4B, and PDE4D protein expression in the rabbit SA node compared to the left ventricle (LV = 100%), column statistics * *p* < 0.05 (from [11]). (**F**) Relative increases in the spontaneous SANC beating rate produced by selective inhibitors of cAMP-degrading PDEs (PDE1-PDE4), compared to broad-spectrum PDE inhibitor IBMX or ISO, and expressed as % of control. One-way ANOVA with Tukey post hoc test, * *p* < 0.05 vs. all PDE inhibition.

The level of cAMP in the cell is determined not only by synthesis by ACs, but by cyclic nucleotide PDEs, which constantly degrade cAMP. High basal level of cAMP in SANCs could be a result of low cAMP degradation and negligible PDE activity in the basal state. On the other hand, high basal PKA- and CaMKII-dependent protein phosphorylation in SANC creates positive feedback system (Figure 3A), which requires strong feedback regulation. Indeed, basal PKA- and CaMKII-dependent phosphorylation promotes Ca^2+^ influx via L-type Ca^2+^ channels, boosts phosphorylation of PLB, and increases SERCA activity, accelerating kinetics of SR replenishment with Ca^2+^. At the same time, an increased RyR phosphorylation synchronizes RyR [74] and likely decreases the threshold for RyR Ca^2+^ release, elevating Ca^2+^ release from the SR via spontaneous LCRs. Higher levels of intracellular Ca^2+^ further stimulate Ca^2+^-activated ACs, which generate more cAMP further activating PKA and CaMKII. This positive feedback system in cardiac pacemaker cells, when Ca^2+^ release creates more Ca^2+^ release, elevating cAMP and amplifying the original action, is unstable and requires robust feedback regulation (Figure 3A), provided by PDEs. Indeed, there is a high basal level of PDE activity in rabbit SANC, since broad-spectrum PDE inhibitor IBMX produces ~9-fold increase in the cAMP level, an effect larger than that of a saturating concentration of β-AR agonist isoproterenol (Figure 3B), [41]. Therefore, constitutively active ACs in the basal state coexist with high basal PDE activity in SANCs, and the latter perform a negative feedback regulation to limit and fine-tune the basal cAMP level.

PDEs represent a highly diverse superfamily of enzymes encoded by 21 genes and divided into 11 families that give rise to over 100 PDE isozymes [75,76]. Three PDE families, PDE4, PDE7, and PDE8 specifically hydrolyze cAMP, three PDE families PDE5, PDE6, and PDE9 hydrolyze cGMP and five PDE families PDE1–PDE3, PDE10, and PDE11 hydrolyze both cAMP and cGMP. Although numerous PDEs, except PDE6 and PDE10, were found in myocardial tissue of different species, including humans, PDE1, PDE3, and PDE4 families are the main PDEs that hydrolyze cAMP in the heart [77]. While PDE3 family dominates in larger mammals including dog, bovine, rabbit, and human myocardium, PDE4 predominates in rodent myocardium [77]. Though PDE4 is also expressed in the human heart, it accounts for only ~10% of the total cAMP-PDE activity (vs. 40–60% in rat and mouse) [77,78]. 

PDE1 is Ca^2+^/calmodulin-activated isoenzyme, hydrolyzing both cAMP and cGMP with similar substrate specificity, and PDE1 subfamily includes three genes (PDE1A, PDE1B, and PDE1C). Though PDE1 isoforms are highly expressed and have high activity in human myocardium [79,80], the physiologic role of PDE1 isoforms in the heart remains unclear, since inhibition of PDE1 activity produces a decrease rather than increase in contraction amplitude of human ventricular myocytes [80]. Experiments in cell culture suggest that PDE1 may not be active under basal conditions but becomes active when intracellular [Ca^2+^]_i_ concentration is increased [81].

PDE2 is cGMP-activated PDE and it can hydrolyze both cAMP and cGMP. Although PDE2 exhibits similar cAMP and cGMP substrate specificity in rat, guinea pig, and dog ventricular tissue, it preferentially hydrolyses cGMP in rabbit, porcine and human myocardium [77]. PDE2 is considered to act primarily as a signal integrator between cGMP and cAMP signaling, and most studies support the idea that under normal conditions PDE2 is responsible for relatively small fraction of total cAMP hydrolyzing activity in the myocardium [75].

PDE3 family is divided in two subfamilies PDE3A and PDE3B, and both are expressed in the heart of different species [77]. Genetic manipulations of mice demonstrated that PDE3A modulates basal excitation-contraction coupling, SR Ca^2+^ content and contractility in cardiac myocytes [77,82]. Ablation of PDE3A eliminated >85% of the PDE3 activity and increased contractility in hearts from mice lacking PDE3A, but not PDE3B. The enhanced cardiac contractility in (PDE3A KO) hearts was associated with phosphorylation of key proteins involved in the regulation of SR Ca^2+^ cycling in cardiac myocytes PLB and RyR [83]. Specifically, there was a 2-fold increase in PLB phosphorylation at PKA-dependent Ser^16^ site accompanied by a 2-fold increase in RyR phosphorylation at PKA-dependent Ser^2808^ and Ser^2830^ sites [83]. The role of PDE3A in the modulation of the SR Ca^2+^ cycling protein phosphorylation is likely linked to its regulation of cAMP in microdomains containing macromolecular complexes of SERCA2a-PLN-PDE3A [83,84].

The PDE4 family consists of four subfamilies, PDE4A to PDE4D, but only PDE4A, PDE4B, and PDE4D are expressed in rodents’ hearts [85]. Although PDE4 has little effect in the resting state, it becomes active upon β-AR stimulation and starts to regulate global cAMP level in cardiac cells. For example, pharmacological inhibition of PDE4 in rat ventricular myocytes increases inotropic effects of β-AR stimulation and spontaneous diastolic Ca^2+^ waves [86]. Moreover, inhibition of PDE4 in the presence of isoproterenol potentiates phosphorylation of RyR2 and PLB, not only at specific PKA-dependent sites (RyR at Ser^2808^ and PLB at Ser^16^), but also at CaMKII-dependent sites (RyR Ser^2814^ and PLB at Thr^17^), increasing SR Ca^2+^ load and SR Ca^2+^ leak [86]. 

Genetic ablation of PDE4D enhanced the susceptibility to stress-induced ventricular tachycardia, which was explained by PKA-dependent hyperphosphorylation of RyR2 [69]. Baseline cardiac contractility in PDE4D-KO mice was markedly elevated and cardiomyocytes isolated from PDE4D-KO hearts showed increased Ca^2+^ transient amplitudes with preserved I_Ca,L_, compared to WT cardiomyocytes [87]. These functional changes in PDE4D-KO myocardium were associated with increased PLB phosphorylation likely due to association of PDE4D with the PLB-SERCA2A complex. Both PDE4B and PDE4D can associate with the L-type Ca^2+^ channel, but only PDE4B regulates I_Ca,L_ during β-AR stimulation in mouse ventricular myocytes. It was concluded that PDE3 and PDE4 families represent major PDE families to degrade cAMP and regulate excitation-contraction coupling in the myocardium [77,78], with PDE3 dominating in larger mammals and in humans [77,78]. 

Comparison of the expression levels of PDE mRNA in isolated rabbit SANC and ventricular myocytes demonstrated that PDE3A, PDE4A, PDE4B, and PDE4D are the major cAMP degrading PDE subtypes expressed in both rabbit SANC and ventricular myocytes [11] (Figure 3C). Though expression of PDE1 mRNA in rabbit SANC was relatively low, PDE1A transcript abundance in SANC by 4-fold surpassed that in ventricular myocytes [89]. Expressions of PDE3A and PDE4B mRNA in rabbit SANC were markedly higher than expression of other PDE subtypes (Figure 3C), while in the mouse SA node mRNA transcript abundance for PDE2A, PDE3A, PDE4A, PDE4B, and PDE4D were similar (Figure 3D) [88]. At the protein level, expression of PDE3A and PDE4A protein was less abundant in the rabbit SA node compared to the left ventricle; expression of PDE4B protein was similar in both tissues, while expression of PDE4D [11] and PDE1A protein [89] was significantly higher in the rabbit SA node than in the ventricle (Figure 3E). 

Compared to the effect of broad-spectrum PDE inhibitor IBMX or saturating concentration of β-AR agonist ISO, the increase in the spontaneous beating rate produced by selective inhibitors of PDE1, PDE2 or PDE3 activity was relatively small, while inhibition of PDE4 had no noticeable effect on spontaneous SANC firing (Figure 3F). Despite elevated basal activity of PDE1 in rabbit SANC [89], PDE1 inhibitor MIMX increased spontaneous firing of rabbit SANC by ~15% (Figure 3F). It is possible that PDE1 activity might have a greater impact at higher cAMP levels; e.g., stimulation of ACs with forskolin markedly increases both the cAMP level and PDE1 activity in paced mouse ventricular myocytes, suggesting that the contractility-coupled Ca^2+^ pool can activate PDE1 [90]. 

Effects of broad-spectrum PDE inhibition on the increase in cAMP level (Figure 3B) and the spontaneous SANC beating rate (Figure 3F) exceeded those of the saturating concentration of β-AR agonist isoproterenol (*p* < 0.05) [41], likely due to the more efficient cAMP degradation by PDEs in the basal state compared to cAMP production triggered by β-AR stimulation. Moreover, data in Figure 3F indicate that normal automaticity of cardiac pacemaker cells is likely regulated, not by one individual PDE subtype, but combined activity of several PDEs. 

## 6. Basal Spontaneous Firing of Rabbit SANC Is Regulated by Dual (PDE3 + PDE4) Activation

Previous studies in a variety of cell types indicated that while inhibition of PDE3 or PDE4 alone have relatively small effects on their own, combined PDE3 + PDE4 inhibition could produce a large synergistic response, creating effect which is greater than the simple sum of separate PDE3 and PDE4 inhibition [7,14]. For example, under basal conditions, effects of PDE3 or PDE4 inhibitor alone on lipolysis or glucose uptake or uncoupling protein-1 expression were relatively small, and substantial effect was reached only with combination of PDE3 + PDE4 inhibitors, which synergistically stimulated each of these processes [7]. Multiple physiological functions as diverse as regulation of smooth muscle cell motility [8] or excitation-contraction coupling in rat ventricular myocytes [9] or right atrium contractility [10] are also regulated by dual (PDE3 + PDE4) activation.

**Figure 4:**
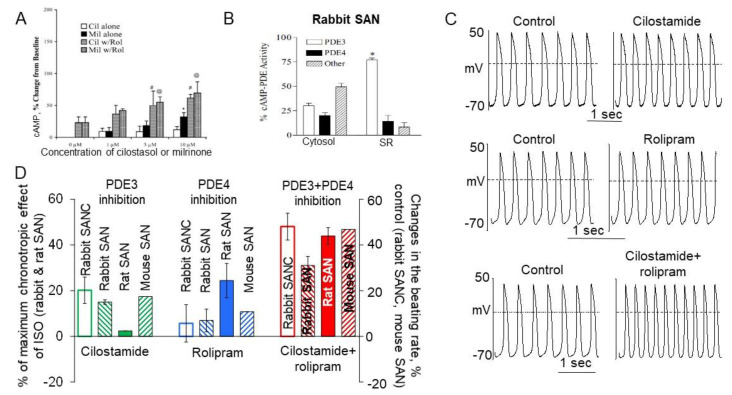
Dual PDE3 + PDE4 inhibition increases spontaneous beating rate of isolated SANC and intact SA nodes in a synergistic manner. (**A**) Inhibition of PDE4 with rolipram (1 µmol/L) in combination with PDE3 inhibitors cilostazol or milrinone (3 or 10 µmol/L) causes synergistic increases in cAMP levels in rabbit ventricular myocytes. # *p* < 0.05 vs. the additive effects of rolipram and cilostazol alone. @ *p* < 0.05 vs. the additive effects of rolipram and milrinone alone (modified from [91]). (**B**) Distribution of PDE3 and PDE4 activities in SR-enriched and cytosolic fractions in rabbit SA node, * *p* < 0.001 vs. PDE4 or ‘other’ PDE activity (**A**,**B** with permission from [91]). (**C**) Representative recordings of APs of intact rabbit SANC prior to and during inhibition of PDE3 (top, cilostamide 0.3µmol/L) or PDE4 (middle, rolipram 2 µmol/L) alone or dual PDE3 + PDE4 inhibition (bottom) (from [11]). (**D**) The positive chronotropic effect produced by PDE3 or PDE4 inhibition alone or dual PDE3 + PDE4 inhibition in isolated rabbit SANC (cilostamide 0.3 µmol/L and rolipram 2 µmol/L, from [11]) or intact rabbit SA node (cilostamide 0.3 µmol/L and rolipram 10 µmol/L; graphic modification from [93]) or intact mouse SA node (cilostamide 0.3 µmol/L and rolipram 1 µmol/L; graphic modification from [94]) or intact rat SA node (cilostamide 0.3 µmol/L and rolipram 1 µmol/L; graphic modification from [95]).

Several studies investigated effects of separate or concurrent PDE3 and PDE4 inhibition on intracellular levels of cAMP in cardiomyocytes. PDE3 inhibitors cilostazol or milrinone produced modest dose-dependent increase in intracellular cAMP in rabbit ventricular myocytes, but dual inhibition of PDE3 + PDE4 caused synergistic elevation of the cAMP level [91] (Figure 4A). In isolated pig cardiomyocytes with expressed cAMP sensor Epac-S^H187^ PDE3 inhibitor cilostamide or PDE4 inhibitor rolipram slightly, but significantly (<20%) increased the CFP/YFP ratio to similar levels [92]. Dual inhibition of PDE3 and PDE4, however, resulted in substantial cAMP elevation by 130.2 ± 13.9%, indicating synergistic effect of concomitant PDE3 + PDE4 activation on basal cAMP level [92].

PDE3 and PDE4 represent the major cAMP-degrading PDE activities in the rabbit SA node [91], i.e., their combined activity in cytosolic or SR fraction accounts for ~50% and ~90% of total cAMP–PDE activity respectively (Figure 4B), while contribution from other PDE subtypes is relatively small. It has been later reported that nimodipine-sensitive activity of PDE1, measured in lysates of rabbit SANC, accounted for ~40% of total PDE activity (likely in cytosolic fraction) [89], but effects of PDE1 inhibition on the spontaneous SANC beating rate are relatively small (Figure 3F). 

Inhibition of PDE3 alone increased DD rate and spontaneous firing of rabbit SANC by ~30% and ~20% (*p* < 0.05) respectively, while effects of PDE4 inhibitor rolipram on both parameters did not reach statistical significance (Figure 4C,D). Concurrent inhibition of PDE3 + PDE4 markedly increased the DD rate and spontaneous SANC beating rate by ~70% and ~48% (*p* < 0.01) respectively, [11] (Figure 4C,D). An acceleration of spontaneous SANC firing by dual PDE3 + PDE4 inhibition by ~2-fold exceeded the sum of increases in spontaneous firing produced by inhibition of PDE3 (~20%) and PDE4 (~5%) alone and was comparable to the effect of broad-spectrum PDE inhibitor IBMX. These results demonstrate that concurrent PDE3 + PDE4 activation regulates normal beating rate of cardiac pacemaker cells in a synergistic manner. The results in isolated rabbit SANC were consistent with observations in the isolated rabbit, rat, or mouse SA nodes [93,94,95]. The effects of PDE3 or PDE4 inhibition alone on the spontaneous beating rate of the isolated rabbit or mouse SA nodes were similar to those of rabbit SANC: PDE3 inhibition increased spontaneous firing (<20%) while PDE4 had almost no effect (Figure 4D). In the rat SA node, however, only inhibition of PDE4 increased spontaneous firing by ~24%, while inhibition of PDE3 had almost no effect (Figure 4D). Nonetheless, concurrent inhibition of PDE3 + PDE4 increased spontaneous firing of the isolated rabbit, rat, or mouse SA nodes in the range of ~30–47% (Figure 4D), which surpassed added increases in the spontaneous beating rates produced by inhibition of PDE3 and PDE4 alone by at least ~1.5-fold. These results demonstrate that, like in rabbit SANC, dual PDE3 + PDE4 activation operates synergistically to regulate basal spontaneous beating rate of isolated SA nodes of various species. The robust modulation of the basal spontaneous beating rate of the cardiac pacemaker by dual PDE3 + PDE4 activation both at the cell and tissue levels could be accomplished only through synchronized PDE3 and PDE4 effects on specific targets of the coupled-clock system (Figure 1), which are discussed below.

## 7. Synergistic Effect of Dual PDE3 and PDE4 Inhibition on L-type Ca^2+^ Current in SANC and Atrial/ventricular Myocytes

**Figure 5:**
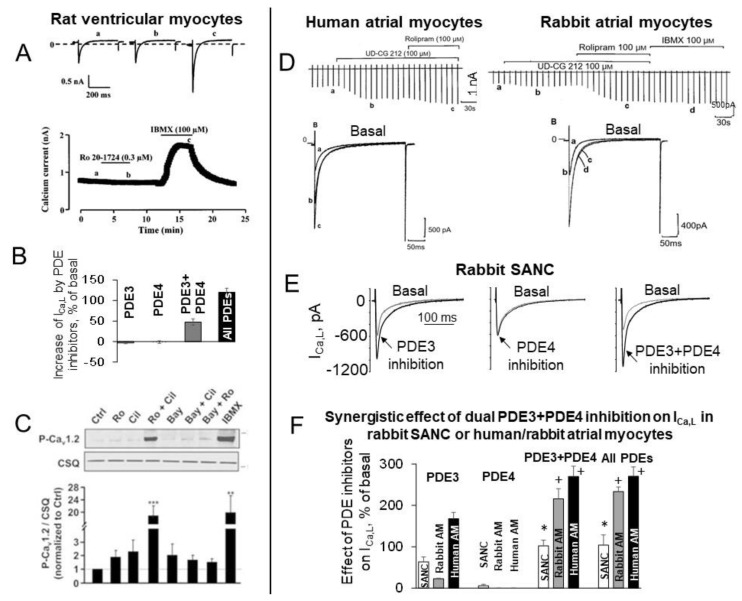
Synergistic regulation of basal L-type Ca^2+^ current amplitude by dual PDE3 + PDE4 activation in rat ventricular myocytes, human, or rabbit atrial myocytes and rabbit SANC. (**A**) Time-dependent effects of PDE4 inhibitor Ro20-1724 and IBMX on I_Ca,L_ in a rat ventricular myocytes (with permission from [97]). (**B**) Both dual PDE3 + PDE4 inhibition (0.1 µmol/L cilostamide and 0.1 µmol/L Ro20-1724) and IBMX (100 µmol/L) markedly increased I_Ca,L_ in rat ventricular myocytes, while PDE3 or PDE4 inhibitors alone were without effect (calculated from Table 1 from [97]). (**C**) Effects of PDE inhibitors alone or in combination on phosphorylation of Ca_V_1.2 in rat ventricular myocytes in basal conditions: PDE4 (Ro20-1724, 10 µmol/L), PDE3 (cilostamide, 1 µmol/L), PDE3 + PDE4 (Ro20-1724 + cilostamide), PDE2 (Bay 60-7550 0.1 µmol/L), PDE2 + PDE3 (Bay 60-7550 + cilostamide), PDE2 + PDE4 (Bay 60-7550 + Ro20-1724), and IBMX (100 µmol/L). The membranes were stripped and re-probed with calsequestrin (CSQ) antibodies used as a loading control (with permission from [9]). (**D**) Representative examples of the enhanced effect caused by combined application of PDE3 (UD-CG 212) and PDE4 (rolipram) inhibitors on I_Ca,L_ in a human (left) and rabbit (right) atrial myocytes (with permission from [98]). (**E**) Representative recordings of I_Ca,L_ in rabbit SANC in response to inhibition of PDE3 (0.3 µmol/L cilostamide) or PDE4 (2 µmol/L rolipram) alone or dual PDE3 + PDE4 inhibition (from [11]). (**F**) Average effects produced by inhibition of PDE3 or PDE4 alone or dual PDE3 + PDE4 inhibition in rabbit SANC (from [11]) and human or rabbit atrial myocytes (from [98]). One-way ANOVA with Tukey post hoc test adjusted, * *p* < 0.05 (rabbit SANC: PDE3 + PDE4 or IBMX vs. PDE3 or PDE4 alone); + *p* < 0.001 (rabbit or human AM: PDE3 + PDE4 or IBMX vs. PDE3 or PDE4 alone).

It is well established that I_Ca,L_ in cardiac myocytes is modulated by cAMP-mediated PKA-dependent phosphorylation: when L-type Ca^2+^ channels are phosphorylated, the open probability of individual channels is increased leading to an increase in I_Ca,L_ amplitude [96]. L-type Ca^2+^ channels are indirectly regulated by PDEs via cAMP-PKA-signaling, and PDE inhibition by IBMX creates marked increases in the I_Ca,L_ amplitude in cardiac myocytes of various species, including guinea pigs, rats, mice, rabbits, and humans (for review see [77]). An impact of different PDE isoforms on cAMP-PKA-mediated modulation of I_Ca,L_, however, is not identical in different regions of the heart. For example, in rat ventricular myocytes inhibition of PDE1, PDE2, PDE3, or PDE4 had no effect on basal I_Ca,L_ amplitude and relatively small effect on cAMP level [97]. A marked stimulation of basal I_Ca,L_ amplitude was produced by IBMX, which increases basal I_Ca,L_ by ~120% (Figure 5A,B) and [cAMP]_i_ by ~70%, or dual inhibition of PDE3 and PDE4, which increases basal I_Ca,L_ by ~50% (Figure 5B) [97]. These results were further verified by a recent study from the same group, which showed marked increases in the phosphorylation of L-type Ca^2+^ channels in rat ventricular myocytes by dual PDE3 + PDE4 or broad-spectrum PDE inhibition (Figure 5C). In contrast, selective PDE2, PDE3, or PDE4 inhibitors alone as well as dual PDE2 + PDE3 or PDE2 + PDE4 inhibition had no effect on phosphorylation of L-type Ca^2+^ channels in the basal state (Figure 5C) [9].

Broad-spectrum PDE inhibitor, IBMX, markedly increased basal I_Ca,L_ amplitude by ~130%, ~140% and ~185% in different regions of the mouse myocardium, including ventricular, atrial myocytes, and SANC, respectively [88]. Consistent with previous results [97], none of the selective PDE2, PDE3, or PDE4 inhibitors had any effect on basal I_Ca,L_ amplitude in mouse ventricular myocytes. In mouse atrial myocytes, however, selective PDE2 or PDE4 inhibitors increased basal I_Ca,L_ by ~38% and ~72%, respectively, while PDE3 inhibition was without effect [88]. In mouse SANC inhibition of PDE2, PDE3, or PDE4 increased I_Ca,L_ by ~31%, ~66%, and ~93%, respectively [88], confirming differences between ventricular, atrial myocytes, and SANC in terms of whether PDEs regulate I_Ca,L_ amplitude in the basal state and, if so, what specific PDE isoforms modulate I_Ca,L_ in cells from different regions of the heart. Dual PDE3 and PDE4 inhibition markedly increased basal I_Ca,L_ in mouse ventricular myocytes by ~50%, while effect on I_Ca,L_ in mouse atrial myocytes or SANC was markedly higher and comparable to that of IBMX [88]. 

In human atrial myocytes, only a small increase in the basal [cAMP]_i_ level was observed in response to PDE4 inhibition alone, while an increase in [cAMP]_i_ was 2-fold larger in response to PDE3 inhibition and 4-fold larger when both PDE3 and PDE4 were concurrently inhibited [99], indicating that concurrent activation of PDE3 and PDE4 controlled basal cAMP level in human atrial myocytes in a synergistic manner. PDE4 inhibition in a concentration-dependent manner moderately increased basal I_Ca,L_ amplitude, which was further elevated by PDE3 inhibition [99]. Another study, however, failed to find any effect of PDE4 inhibitor rolipram on basal I_Ca,L_ amplitude in human atrial myocytes, likely due to differences in the experimental conditions [100].

There was substantial increase in the basal I_Ca,L_ amplitude in response to PDE3 inhibitor in rabbit and especially human atrial myocytes, but not to PDE4 inhibitor in both species [98]. Dual PDE3 + PDE4 inhibition, however, increased I_Ca,L_ by ~215% and ~270% in human or rabbit atrial myocytes respectively [98], creating synergistic amplification of I_Ca,L_ amplitude. The effect of dual PDE3 + PDE4 inhibition markedly exceeded additive effects of PDE3 or PDE4 inhibition alone (Figure 5D,F) and was comparable to the effect of IBMX [98]. L-type Ca^2+^ channels are essential for spontaneous firing of cardiac pacemaker cells, and they are part of both “membrane clock” and “Ca^2+^ clock”: L-type Ca^2+^ current generates AP upstroke in primary pacemaker cells and at the same time provides Ca^2+^ supply to replenish the SR Ca^2+^ store boosting LCR generation. Similar to atrial myocytes, basal PKA-dependent phosphorylation also regulates L-type Ca^2+^ channels in rabbit SANC, i.e., PKA inhibitor peptide PKI suppresses basal amplitude of I_Ca,L_ in SANC by ~80% [49]. Similar to human or rabbit atrial myocytes, inhibition of PDE4 alone in rabbit SANC had no effect on I_Ca,L_ amplitude, while inhibition of PDE3 increased I_Ca,L_ by ~60% [11] (Figure 5E,F). This effect was further amplified by dual PDE3 + PDE4 inhibition, which increased I_Ca,L_ amplitude in rabbit SANC by ~100% [11] (Figure 5E,F). Therefore, dual PDE3 + PDE4 activation regulates basal I_Ca,L_ amplitude both in rabbit SANC and human/rabbit atrial myocytes in a synergistic manner, creating effect that markedly exceeds added effects of PDE3 or PDE4 activation alone (Figure 5F). 

In excitable cells, L-type Ca^2+^ channels could regulate AC activation, e.g., AC8 when expressed in pancreatic cells is stimulated, while the endogenous AC5/AC6 in cardiomyocytes are inhibited by I_Ca,L_ [101]. Immunostaining studies demonstrated similar subcellular localization of AC5/AC6 with L-type Ca^2+^ channels in ventricular myocytes [102]. Since ACs have scaffolding properties [103] and can bind AKAPs [104], by sharing AKAPs, they can form a complex with L-type Ca^2+^ channels. Indeed, Ca^2+^-inhibited AC5 and AC6 can bind L-type Ca^2+^ channels indirectly by sharing AKAP79 [105]. The AC5 isoform is localized mainly in caveolin-reach regions of t-tubules of mouse ventricular myocytes where its effect on I_Ca,L_ is strongly modulated by dual PDE3 and PDE4 activation [106]. In contrast, the AC6 isoform is associated with the plasma membrane (outside t-tubular region) and is responsible for an increase of I_Ca,L_ during β1-AR stimulation [106]. Not only Ca^2+^ influx through L-type Ca^2+^ channels can modulate AC activation, but ACs also can regulate I_Ca,L_ amplitude. In guinea pig atrial myocytes, Ca^2+^-stimulated AC1 and AC8 are present and functionally active in the basal state, and activation or inhibition of ACs is paralleled by an increase or decrease of the L-type Ca^2+^ current amplitude [107].

**Figure 6:**
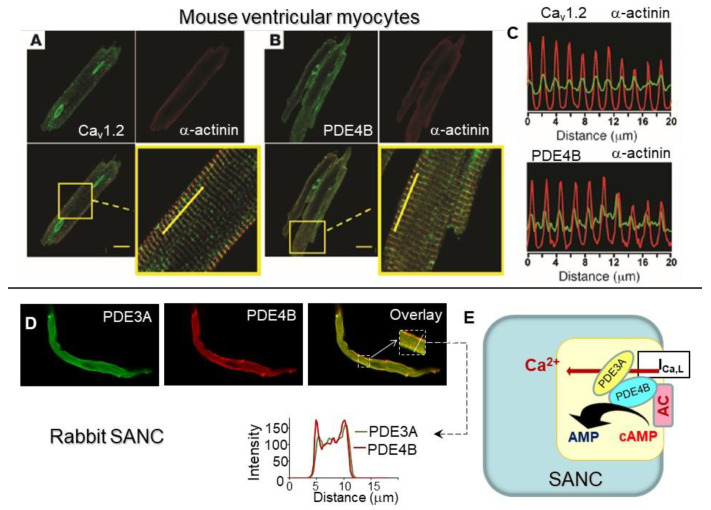
Co-localization of PDE4B with L-type Ca^2+^ channels in ventricular myocytes and SANC. (**A**–**C**) (with permission from [85]): (**A**) Localization of PDE4B and L-type Ca^2+^ channels at the transverse tubules (t-tubules) in mouse ventricular myocytes. Confocal images of WT mouse ventricular myocytes double labeled with anti-Ca_V_1.2 antibody (green) and anti-α-actinin antibody (red). (**B**) Confocal images of a WT mouse ventricular myocytes double labeled with anti-PDE4B antibody (green) and anti-α-actinin antibody (red). Scale bar: 20 µm. (**C**) Graphs indicate the relative fluorescence intensities in the green and red channels measured along a 20-µm distance, as indicated by the yellow line on the enlarged boxed area from the merged images. (**D**) Distribution of PDE3A and PDE4B in single rabbit SANC, which do not have t-tubules: green fluorescence staining for PDE3A and red fluorescence staining for PDE4B (negative control had negligible fluorescence). Superimposed images (Overlay) and intensity plot (below) show overlapping distribution of PDE3A with PDE4B beneath sarcolemma (from [11]). (**E**) Cartoon shows plausible PDE3/4-dependent regulation of AC-cAMP signaling in the vicinity of L-type Ca^2+^ channels in SANC.

In the mouse heart, PDE4B has been recognized as a part of the L-type Ca^2+^ channel complex and represents the major PDE isoform modulating I_Ca,L_ amplitude during β-AR stimulation [85]. In ventricular myocytes, L-type Ca^2+^ channel signaling complex is located in t-tubules and co-localized with PDE4B in the striated pattern with the peak intensity band overlapping α-actinin, which is also located in t-tubules (Figure 6A–C). Altogether, colocalization of Ca_V_1.2 and PDE4B along t-tubule membranes in the mouse heart strongly suggests that L-type Ca^2+^ channels represent the primary target of PDE4B. 

Immunostaining of rabbit SANC demonstrated that part of PDE3A is located beneath sarcolemma (Figure 6D), and co-staining of PDE3A and PDE4B showed higher labeling intensity in the overlay images beneath sarcolemma of SANC (see intensity plots in the bottom panel of Figure 6D). These results show that part of PDE3A and PDE4B are co-localized beneath sarcolemma of SANC, and it is likely that these PDEs, working as a team, limit Ca^2+^ influx through L-type Ca^2+^ channels in a synergistic manner (Figure 5F and Figure 6E). ACs reside within lipid microdomains of SANC (see chapter above), AC1 and, to lesser extent, AC8 immunolabeling closely tracks that of caveolin and Ca^2+^-dependent activation of AC activity also occurs within these domains [42]. Therefore, functional L-type Ca^2+^ channels, ACs, PDE3A, and PDE4B are all distributed in the close proximity of the sarcolemma of SANC, suggesting that PDE3A, PDE4B, and ACs could be part of L-type Ca^2+^ channel signaling complex (Figure 6E). This idea, however, requires further verification.

## 8. PDE3 and PDE4 Regulate Basal SR Ca^2+^ Cycling in Cardiac Myocytes and SANC

**Figure 7:**
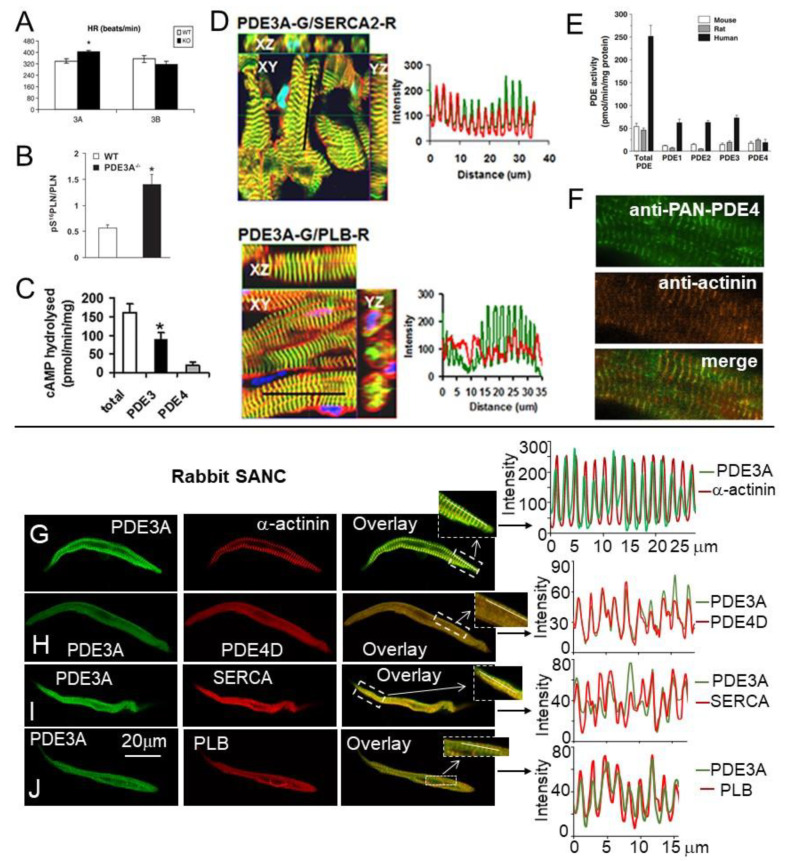
Distribution of PDE3 and PDE4 in human ventricular myocytes and rabbit SANC. (**A**) Baseline value of heart rate (HR) in PDE3A-KO and matched WT, and PDE3B-KO and WT mice (with permission, from [82]). (**B**) Increased levels of PKA- dependent phosphorylation of PLB at Ser^16^ site in PDE3A-KO hearts (with permission, from [83]). (**C**) PDE activity in human cardiac SR fractions: PDE3 activity was significantly higher than PDE4 activity (* *p* < 0.001) (with permission, from [108]). (**D**) PDE3A, SERCA2, and PLB co-localize in the Z-bands in normal human myocardium. Merged images from stacks of 10–15 sections (with 1-µm intervals) reveal colocalization of PDE3A with SERCA2 and PLB (with permission, from [108]). (**E**) Expression of PDE subtypes in mouse, rat, and human heart presented as specific PDE activity in pmol/min/mg (with permission, from [78]). (**F**) Cryosections of human myocardium were double-stained with antibodies against sarcomeric actinin (red) and anti-PAN-PDE4 antibodies (green) show that PDE4 localizes to the Z-band of cardiomyocytes in human heart (with permission, from [78]). (**G**–**J**) From [11]; from the left: first column, green fluorescence staining for PDE3A; second column, red fluorescence staining for marker proteins: (**G**) α-actinin, (**H**) PDE4D, (I) SERCA2, and (**J**) PLB, respectively; third column merged images of PDE3A and marker proteins in panels (**G**–**J**). Insets in overlay show magnification of the rectangular areas in panels (**G**–**J**). Fourth column: intensity plots calculated along white lines in dashed rectangles. Superimposed images (overlay) and intensity plots showed overlapping distribution of PDE3A with α-actinin, PDE4D, SERCA, and PLB along Z-lines; negative controls had negligible fluorescence.

Cyclic nucleotide PDEs are essential enzymes degrading cAMP to regulate basal cardiac function, including resting beating rate of the heart and cardiac contractility. Studies in genetically modified mice established that chronotropic and inotropic responses to PDE3 inhibition are present in PDE3B-KO mice, but not in PDE3A-KO mice, indicating that PDE3A isoform is essential for regulation of both the heart-beating rate and cardiac contractility [82]. Specifically, the heart rate of PDE3A KO mice was significantly higher, compared with WT mice, while that of PDE3B KO mice remained similar to WT mice [82] (Figure 7A). The enhanced contractility in PDE3A-KO hearts was linked to the increased PLB phosphorylation at the PKA-dependent Ser^16^ site (Figure 7B), subsequent elevation of SERCA activity and SR Ca^2+^ content, which led to increased Ca^2+^ transient amplitudes in cardiac myocytes without changes in L-type Ca^2+^ currents [83]. Moreover, there was a substantial increase in the RyR2 phosphorylation at both PKA-dependent Ser^2808^ and Ser^2830^ sites and the CaMKII-dependent Ser^2814^ site, accompanied by reduced RyR2 expression [83]. The matching changes in PLB and RyR2 phosphorylation suggest that PDE3A regulates cAMP at the local level in the SR regions of cardiac myocytes, controlling at once both Ca^2+^ uptake and Ca^2+^ release. Immunoprecipitation studies, however, demonstrated that PDE3A colocalizes with SERCA2 and PLB, but not with RyR, suggesting that regulation of cAMP levels by PDE3A at the vicinity of SERCA2 may have extending effects to the adjacent RyR2 channels. In mouse myocardium, PDE3A isoform was found to be a part of SERCA2-PLB-AKAP18 multiprotein complex or “signalosome” that regulates the transport of Ca^2+^ into SR [83]. 

PDE3 represents the major PDE activity in human cardiac myocytes (Figure 7C), while PDE4 activity is relatively low. In human SR fractions, cAMP increased PLB phosphorylation at Ser^16^ site and, as a result, SERCA2 activity; this effect was further potentiated by PDE3 inhibition, but not by PDE4 inhibition [108]. Data in mouse myocardium were consistent with results in human cardiac myocytes, which demonstrated colocalization of PDE3A with desmin, SERCA2, PLB, and AKAP18 [108] (Figure 7D), supporting the idea that PDE3A is distributed in a striated pattern in Z-lines of human cardiac myocytes, and regulates cAMP levels within microdomains of SR containing a macromolecular signaling complex formed of SERCA2 with its major regulatory partners [108,109]. Moreover, PKA-dependent phosphorylation of PDE3A increases its cAMP-hydrolytic activity and facilitates its incorporation in SERCA2/AKAP18 signalosome where it regulates local cAMP pools that control cardiac contractility by modulating PLB phosphorylation and SERCA2 activation [108]. 

In contrast to PDE3, the effects of acute PDE4 inhibition on cardiac performance, including beating rate and contractility, in many species are subtle. Using transgenic mice, it was discovered that PDE4D regulates baseline SR Ca^2+^ release and cardiac contractility in a PI3Kγ-dependent manner [87]. Compared to WT mice, PDE4D-KO mice have augmented myocardial contractility, which is associated with increased contraction of cardiac myocytes, increased Ca^2+^ transient amplitudes and SR Ca^2+^ content, without changes in I_Ca,L_ [87]. The functional changes in PDE4D-KO myocardium were linked to increased PLB phosphorylation, but not RyR2 receptor phosphorylation. A previous study, however, found that PDE4D3 is part of macromolecular cardiac RyR2 signaling complex [69], and PDE4D-KO mice develop heart dysfunction and arrhythmias, resulting from RyR2 hyperphosphorylation at Ser^2808^. The discrepancies between two studies may be related to different reagents or to the age-dependent changes in cAMP regulation and require further verification. 

In the human heart, total cAMP-hydrolytic activity is five-fold higher compared to rodents [78] due to significantly higher expression of PDE1, PDE2, and PDE3, while expression of PDE4 remains the same (Figure 7E). Therefore, PDE4 inhibition increases phosphorylation of multiple PKA targets in mouse cardiac myocytes, but only a few targets in the human cardiac myocytes [78]. In humans, however, PDE4 is tethered to similar locations as in the rodent heart including β1-AR and SERCA2-PLB macromolecular signaling complexes [78]. Despite the small quantity, PDE4 accounts for ~50% of total cAMP-hydrolytic activity co-localized with PLB, suggesting that PDE4 is an essential PDE that fine-tunes cAMP content and SERCA activity in the SERCA-PLB compartment [78]. The presence of PDE4 in this signaling complex is also consistent with subcellular localization of PDE4 to Z-lines of human cardiac myocytes [78] (Figure 7F). Since PDE3A in human cardiac myocytes is also tethered to Z-lines and SERCA-PLB complex both PDE3A and PDE4 are likely positioned at the same subcellular compartment containing SERCA2-PLB multiprotein signalosome. Studies in transgenic mice and human myocardium confirmed that both PDE3A and PDE4D likely associate with macromolecular complex of the SR including SERCA2 with its major regulatory partners, which play a principal role in regulation of cardiac contractility [78,83,87,99,108].

Immunostaining of rabbit SANC demonstrated that part of PDE3A isoform is distributed beneath sarcolemma (Figure 6D) as well as in a striated pattern colocalized with Z-line associated protein α-actinin (Figure 7G), which did not rely on the presence of t-tubules that are absent in rabbit SANC. Intensity plots in the right panel show that intensity peaks of PDE3A and α-actinin overlap with an interval of ~1.80 ± 0.0 µm. Co-staining of PDE3A with anti-PDE4D antibody discovered colocalization of these PDEs in a striated pattern inside SANC (Figure 7H), which also coincided with the distribution of Z-line associated protein α-actinin (Figure 7G). Co-staining of PDE3A with antibodies for SR associated proteins SERCA and PLB demonstrated colocalization of PDE3A with these major SR Ca^2+^ cycling proteins (Figure 7H–J). Since PDE4D is co-localized with PDE3A, it positions PDE4D in the proximity of SERCA and PLB in rabbit SANC. Consequently, PDE3A and PDE4D in rabbit SANC are co-localized with each other as well as with SERCA and PLB complex suggesting that, similar to mouse or human cardiac myocytes (Figure 7D,F), these PDEs could regulate cAMP-signaling and PKA-dependent phosphorylation in SERCA-PLB compartment in SANC. Therefore, there is a strong preservation of expression patterns of PDE3 and PDE4 with respect to PDE subtypes among different cell types (ventricular myocytes or SANC) as well as various species, including mice, humans, and rabbits [11,78,83,87,108], as PDE3A and PDE4D are tethered to macromolecular signaling complexes that are known to regulate SR Ca^2+^ cycling and specifically the SERCA-PLB complex.

**Figure 8:**
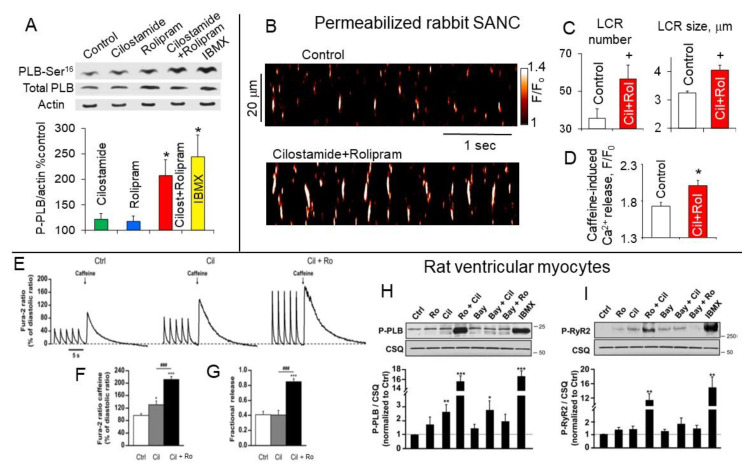
(**A**) Top, representative western blots of total and phosphorylated PLB at PKA-dependent Ser^16^ site in rabbit SANC before and after application of 0.3 µmol/L cilostamide or 2 µmol/L rolipram alone or combination of cilostamide + rolipram or 100 µmol/L IBMX. Bottom, average changes in phosphorylated PLB at Ser^16^ site and expressed as % of control (n = 7–9 rabbits). One-way ANOVA with Tukey’s post-hoc test, * *p* < 0.05 vs. cilostamide or rolipram alone. (**B**) Confocal line-scan images of representative saponin-permeabilized rabbit SANC prior to and during dual PDE3 + PDE4 inhibition. (**C**) Average changes in the LCR number (left) and size (right) produced by 2-min superfusion with 0.3 µmol/L cilostamide and 2 µmol/L rolipram. (**D**) SR Ca^2+^ content was estimated by rapid application of caffeine on permeabilized SANC in the absence or presence of dual PDE3 + PDE4 inhibition. Compared to control group dual PDE3 + PDE4 inhibition markedly increased caffeine-induced SR Ca^2+^ release. *t*-test: + *p* < 0.01; * *p* < 0.05; (**A**–**D**, from [11]). (**E**–**I**, with permission, from [9]). (**E**) Effect of concomitant PDE3 and PDE4 inhibition on SR Ca^2+^ load and fractional release in rat ventricular myocytes. Representative traces of Ca^2+^ transients obtained with caffeine (10 mM) recorded in Fura-2 loaded rat ventricular myocytes after pacing at 0.5 Hz, under basal conditions (Ctrl), upon PDE3 inhibition by cilostamide (Cil, 1 µmol/L), or PDE3 and PDE4 inhibition by cilostamide and Ro20-1724 (10 µmol/L) (Ro + Cil). (**F**) Mean amplitude (±SEM) of calcium transients induced by caffeine (10 mM) estimating SR Ca^2+^ content in Ctrl (white bar, n = 8 cells, 3 rats), Cil (grey bar, n = 8 cells, 3 rats), and Cil + Ro (black bar, n = 9 cells, 3 rats). (**G**) Average fractional release (+SEM) in Ctrl (white bar, n = 8 cells, 3 rats), Cil (grey bar, n = 8 cells, 3 rats), and Cil + Ro (black bar, n = 9 cells, 3 rats). Statistical significance is indicated as: * *p* <0.05; *** *p* < 0.001 (vs. control cells), and ### *p* < 0.001 (vs. Cil treated cells) (one-way ANOVA, Newman–Keuls). (**H**) phospho-PLB (P-PLB) and (**I**) phospho-ryanodine receptor (P-RyR2). The membranes were stripped and re-probed with CSQ antibodies used as a loading control. A representative blot is shown, and at least five separate experiments were performed (n = 6 for P-PLB) and (n = 5 for P-RyR), giving similar results. Phosphorylated proteins/CSQ ratios were quantified, expressed as means ± SEM, and normalized to untreated cells (Ctrl). Statistical significance is indicated as: * *p* < 0.05; ** *p* < 0.01; *** *p* < 0.001 (one-way ANOVA, Newman–Keuls).

Functional significance of PDE3 and PDE4 co-localization in rabbit SANC was tested using phosphorylation status of PLB as a marker for PKA-dependent protein phosphorylation. Inhibition of PDE3 or PDE4 alone moderately increased PLB phosphorylation by ~21% (*p* > 0.05) and ~17% (*p* > 0.05) respectively, but dual PDE3 + PDE4 inhibition increased PLB phosphorylation by ~108% (*p* < 0.05), an effect comparable to that of IBMX, which, by ~2-fold, surpassed additive effects of separate PDE3 or PDE4 inhibition (Figure 8A). To verify whether dual PDE3 + PDE4 activation regulates intrinsic SR Ca^2+^ cycling, avoiding interference of functional ion channels, effects of PDE3 and PDE4 inhibition alone or in combination was studied in saponin-permeabilized SANC. Inhibition of PDE3 alone in permeabilized SANC slightly by ~10% increased the LCR size but no changes in LCR parameters were recorded during inhibition of PDE4 alone [11]. Dual PDE3 + PDE4 inhibition, however, produced synergistic effect and substantially augmented both LCR number by ~60% (*p* < 0.01) and LCR size by ~25% (*p* < 0.01) (Figure 8B,C) exceeding additive effects produced by inhibition of PDE3 or PDE4 alone [11]. The noticeable increase in the SR Ca^2+^ release in permeabilized SANC was due, in part at least, to the substantial increase in the SR Ca^2+^ load [11] (Figure 8D). 

Effects of selective PDE3 and PDE4 inhibitors alone or in combination on SR Ca^2+^ release under basal conditions was studied in rat ventricular myocytes [9]. Selective inhibition of PDE3 induced a small but significant increase in the amplitude of Ca^2+^ transients and sarcomere shortening, whereas PDE4 inhibition had no effect. Concomitant PDE3 + PDE4 inhibition, however, produced synergistic effect, doubled the SR Ca^2+^ load (Figure 8E,F) and considerably increased fractional Ca^2+^ release from 40% in the basal state or after PDE3 inhibition, to 80% when both PDE3 and PDE4 were inhibited (Figure 8G). The effect of concomitant PDE3 + PDE4 inhibition was identical to the effect of the broad-spectrum PDE inhibitor IBMX [9]. Considerable increase in the SR Ca^2+^ load and Ca^2+^ release produced by PDE3 + PDE4 inhibition could be partly explained by the synergistic effect of dual PDE3 + PDE4 inhibition on phosphorylation of proteins involved in the excitation-contraction coupling in ventricular myocytes. Specifically, broad-spectrum PDE inhibitor IBMX or dual PDE3 + PDE4 inhibition substantially augmented phosphorylation of PLB (Figure 8H) and RyR2 (Figure 8I), while inhibition of PDE3 alone or dual PDE2 + PDE3 inhibition only slightly increased PLB phosphorylation (Figure 8H), but not RyR phosphorylation. In contrast, inhibition of PDE2 or PDE4 alone or dual inhibition of PDE2 + PDE4 had no significant effect on phosphorylation of PLB or RyR [9] (Figure 8H,I).

In pig ventricular myocytes, selective inhibition of PDE3 or PDE4 alone induced a slight (<20%) but significant increase in basal cAMP, reported by an increased CFP/YFP ratio [92]. Concomitant inhibition of PDE3 and PDE4, however, resulted in a substantial synergistic cAMP elevation (130.2 ± 13.9%, *p* < 001) [92]. The critical role for PDE3 and PDE4 to control cAMP levels and Ca^2+^ homeostasis was further confirmed by the appearance of spontaneous Ca^2+^ waves (SCWs) upon cessation of pacing during PDE inhibition. Inhibition of PDE3 or PDE4 alone produced only few SCWs, but dual PDE3 + PDE4 inhibition, initiated SCWs in ~80% of cardiomyocytes. These results indicate that both PDE3 and PDE4 are essential to counterbalance basal cAMP synthesis in pig ventricular myocytes, since both PDEs contribute to Ca^2+^ homeostasis and dual PDE3 + PDE4 inhibition leads to a synergistic increase in pro-arrhythmic events [92].

## 9. Synergistic Effect of Dual PDE3 and PDE4 Activation on Local Subsarcolemmal Ca^2+^ Releases in SANC, Role of Compartmentalization

**Figure 9:**
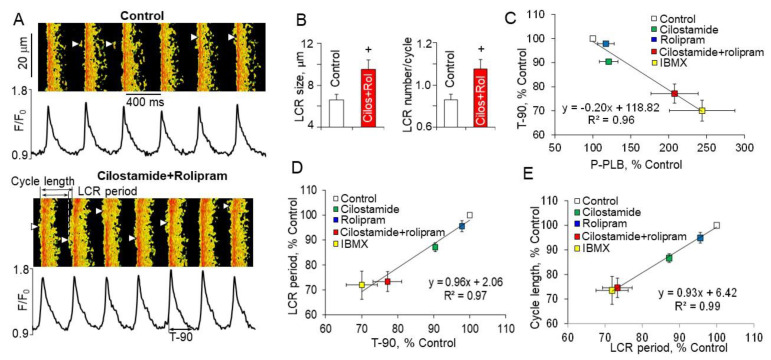
Dual PDE3 + PDE4 activation regulates basal LCR period and characteristics in intact rabbit SANC. (**A**) Confocal line-scan images of representative intact SANC depicting AP-induced Ca^2+^ transients and LCRs (arrowheads) during spontaneous beating: (top) in the basal state and (bottom) during concurrent PDE3 + PDE4 inhibition by 0.3 µmol/L cilostamide and 2 µmol/L rolipram. Normalized subsarcolemmal fluorescence averaged over image width is shown in black beneath the image. Insets in the bottom panel show definitions of the LCR period, spontaneous cycle length and time to 90% decay of AP-induced Ca^2+^ transient (T-90). (**B**) Dual PDE3 + PDE4 inhibition markedly increases (left) the LCR size and (right) LCR number per each spontaneous cycle (n = 7 SANC, 4 rabbits), *t*-test, + *p* < 0.01. (**C**) an increase in PLB phosphorylation produced by inhibition of PDE3 or PDE4 alone or dual PDE3 + PDE4 inhibition is highly correlated with reductions in SR Ca^2+^ refilling times (T-90, n = 4-6 SANC, 3 rabbits) and proportional shortenings of LCR periods (**D**), which predicted (**E**) decrease in spontaneous SANC cycle lengths. (**A**–**E** from [11]).

To elucidate specific mechanisms of the (PDE3 + PDE4)-dependent regulation of basal spontaneous SANC firing effects of PDE3 or PDE4 inhibition alone or dual PDE3 + PDE4 inhibition on LCR period and characteristics were studied in intact rabbit SANC. Inhibition of PDE3 alone markedly increased the LCR size and the number per each spontaneous cycle by ~20% each (*p* < 0.05), while changes in these parameters by rolipram were minor [11]. Dual PDE3 + PDE4 inhibition, however, produced synergistic effect augmenting LCR size and number per each spontaneous cycle by ~40% (*p* < 0.01) each (Figure 9A,B), likely due to an increase in the SR Ca^2+^ content (Figure 8D) and subsequent synchronization of RyR activation via RyR recruitment [67].

During spontaneous beating of intact SANC AP-induced Ca^2+^ transient partially depletes SR and abolishes LCRs, spontaneous LCRs start to occur after the SR Ca^2+^ content is replenished by SERCA [20,110]. The LCR occurrence beneath sarcolemma of SANC activates an inward Na^+^-Ca^2+^ exchange current, which boosts an exponential increase in the late DD rate defining the time of the next AP upstroke (Figure 1). Therefore, the LCR period is a timekeeper of the spontaneous SANC cycle length, which is defined by the rate of SR Ca^2+^ refilling. The velocity of Ca^2+^ pumping into SR by SERCA is modulated by phosphorylation status of PLB [64]. There is a close link between gradations in the increase of PLB phosphorylation during separate or concurrent PDE3 and PDE4 inhibition and reduction in the SR Ca^2+^ refilling times, indexed by the time of the decay of AP-induced Ca^2+^ transient at 90% (T-90) (Figure 9C). Furthermore, the effects of PLB phosphorylation on increased kinetics of SR refilling, indexed by T-90, are reflected in proportional shortenings of the LCR periods (Figure 9D) [110]. 

The graded decreases in LCR periods during inhibition of PDE3 or PDE4 alone or dual PDE3 + PDE4 inhibition are closely correlated with the shortening of the spontaneous SANC cycle lengths (Figure 9E). Thus, concurrent PDE3 + PDE4 activation operates in a synergistic manner to restrict the basal spontaneous SANC beating rate through decrease in cAMP-mediated PKA-dependent protein phosphorylation (indexed by phosphorylation of PLB), suppression of basal LCR parameters and postponement of the LCR occurrence, which delays activation of I_NCX_ and leads to prolongation of the spontaneous SANC cycle length [11]. 

Though synergism between PDE3 and PDE4 isoforms has been noted in different cell types, the mechanisms underlying these synergistic effects remain uncertain. The synergistic effect could be created by colocalization and interaction of different PDE3 and PDE4 subtypes. Indeed, PDE3 and PDE4 have different cAMP affinity: PDE3 affinity is ~10–100 nmol/L [111], while PDE4 affinity is in the range of 2–8 µmol/L [112]. It is possible that PDE3 is active and degrade cAMP in the basal state, while PDE4 remains dormant. Inhibition of PDE3 increases local cAMP and shifts cAMP level within the degradation range of PDE4, switching PDE4 on, and making it crucial to degrade cAMP and regulate cAMP levels on both local and global scales. Moreover, PKA-dependent phosphorylation activates both PDE3 and PDE4 isoforms; PKA-dependent phosphorylation of PDE4 is associated with 2–6-fold increase in PDE4 activity [113]. In human ventricular myocytes, PKA-dependent phosphorylation of endogenous PDE3A increases PDE3A activity and its association with SERCA-PLB-AKAP18 complex in a phosphorylation-dependent manner [108], providing powerful negative feedback for cAMP signals in cardiac myocytes. It is possible that interactions between PDE3 and PDE4 affinities are reinforced and synergized with PKA-mediated activation of both PDEs, thus facilitating negative feedback loop to promote cAMP degradation. In this context, the full functional effect of PDE inhibition could be achieved only when both PDE3 and PDE4 are concurrently inhibited, leading to maximum increase in local levels of cAMP and PKA-dependent phosphorylation and to full-sized functional response. Synchronized regulation of several targets, including L-type Ca^2+^ channels, PLB and likely others, by synergistic dual PDE3 + PDE4 activation could be energetically beneficial since simultaneous changes of local cAMP at the limited number of key locations could lead to substantial functional responses.

Many cellular processes modulated by universal second messenger cAMP require strict control of its range; fine-tuning of cAMP dynamics in space and time is achieved by coordination between AC and PDEs. The fluorescence resonance energy transfer (FRET)-based reporters helped to discover compartmentalized cAMP-signaling (cAMP ‘microdomains’), which restrict signal propagation and permit cAMP to regulate different pathways in a confined manner [114,115,116]. These microdomains are created by scaffolding multiple PDE isoforms, which degrade cAMP at specific locations, preventing cAMP diffusion, and defining borders of individual cAMP pools [29,117,118]. Further development of novel FRET-based cAMP reporters has discovered that differences in local regulation of cAMP may occur within tens of nanometers and, thus, established the existence of spatially restricted subcellular compartments at a nanometer scale, cAMP nanodomains [119]. Such compartments have been revealed for many key proteins that regulate excitation-contraction coupling in cardiac myocytes including L-type Ca^2+^ channels, RyR, and PLB [119,120,121,122]. Each of these proteins is part of a multimolecular complex, which consists of anchored PKA molecules and specific PDE isoforms, which degrade cAMP to terminate and spatially restrict cAMP signaling. 

Comparison of changes in PKA-dependent phosphorylation in SANC, indexed by PLB phosphorylation (Figure 8A), with changes in global cAMP in response to inhibition of PDE3 or PDE4 alone or dual PDE3 + PDE4 inhibition confirmed that dual PDE3 + PDE4 activation likely regulates cAMP and PKA-dependent protein phosphorylation at the local level. Indeed, dual PDE3 + PDE4 inhibition in SANC lysates increased global cAMP significantly less, compared to IBMX or the sum of global cAMP elevations created by inhibition of either PDE3 or PDE4 alone [123]. These results indicate that dual PDE3 + PDE4 activation, operating in a synergistic manner, likely regulates local cAMP pools in the vicinity of PLB or L-type Ca^2+^ channels (scheme in Figure 6E) or RyR and likely others, restricting access of cAMP and limiting PKA-dependent phosphorylation of these essential targets. Despite substantial advances in the field of local microdomain-specific cAMP dynamics in cardiac myocytes, exploration of local cAMP signaling in cardiac pacemaker cells, using FRET-based cAMP reporters, only recently began to emerge [124]. This study was consistent with previous results [40] and showed that kinetics of increases in PKA activity in response to physiological stimuli such as β-AR stimulation or PDE inhibition were tightly linked to changes in the spontaneous SANC firing rate [124]. Future studies that utilize advanced FRET-based cAMP reporters are needed to understand how compartmentalized PDE-dependent signaling and synergistic dual PDE3 + PDE4 activation regulates basal cardiac pacemaker function.

## 10. Funny Current Is Not a Target of Dual PDE3 + PDE4 Activation

**Figure 10:**
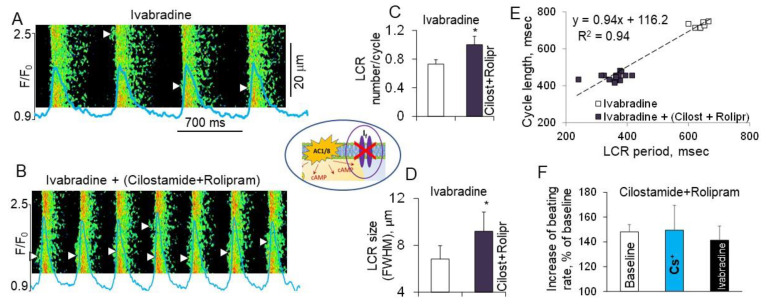
Concurrent (PDE3 + PDE4) inhibition markedly increases LCR parameters, shortens the LCR period and spontaneous SANC cycle length despite suppression of I_f_ current by either ivabradine or Cs^+^. (**A**,**B**) Confocal line scan images of a representative intact rabbit SANC depicting AP-induced Ca^2+^ transients and LCRs (arrowheads) during spontaneous beating of SANC in presence of 5 µmol/L ivabradine (**A**) and (**B**) following inhibition of (PDE3 + PDE4) by 0.3 µmol/L cilostamide and 2 µmol/L rolipram. Normalized subsarcolemmal fluorescence averaged over an image width is shown in blue. (**C**) In the presence of ivabradine, dual (PDE3 + PDE4) inhibition markedly increased the number of LCRs per each spontaneous cycle (left, n = 5 SANC) and (**D**) the LCR size (right, n = 5 SANC); (**E**) (PDE3 + PDE4) inhibition also markedly decreased the LCR period, which was accompanied by the decrease in the spontaneous SANC cycle length. (**F**) Average acceleration of the firing rate by dual PDE3 + PDE4 inhibition presented as % of baseline: in the absence (n = 9 SANC) or presence of either 2 mmol/L Cs^+^ (n = 6 SANC) or 5 µmol/L ivabradine (n = 5 SANC); % of baseline was calculated relative to firing rate before treatment with the combination of cilostamide + rolipram. *t*-test * *p* < 0.05. (**A**–**F**, modified from [11]).

Several ion currents involved in the generation of the DD rate are regulated by PDEs including I_f_ [125], I_K_ [41] and likely others. It is well known that cAMP has a strong impact on the funny current, an important component of the “membrane clock”, by shifting the f-channel activation curve in the positive direction [16,17,126]. Results of several studies in different species demonstrated that I_f_ is also regulated by basal PDE activation. Specifically, broad-spectrum PDE inhibitor IBMX markedly increases I_f_ current amplitude by ~70% [127] and shifts f-channel activation to more positive voltages [125]. PDE3 inhibition significantly by ~20% increases I_f_ current amplitude both in rabbit and mouse SANC [128,129]. These data suggest that the positive chronotropic response produced by dual PDE3 + PDE4 inhibition could be partly due to PDE-dependent modulation of the I_f_ current, and when I_f_ current is inhibited acceleration of the spontaneous beating of SANC by dual PDE3 + PDE4 inhibition might be partly suppressed. To test this idea, the effects of dual PDE3 + PDE4 inhibition were compared in the presence and absence of well-known inhibitor of the I_f_ current ivabradine or Cs^+^, which at 2 mmol/L concentration slightly affects several potassium currents and eliminates I_f_ [130,131]. Figure 10 shows that ivabradine decreased the spontaneous SANC beating rate, but concurrent (PDE3 + PDE4) inhibition markedly increased LCR parameters, decreased the LCR period and this was accompanied by a reduction of the spontaneous SANC cycle length. Overall, the positive chronotropic effect of dual PDE3 + PDE4 inhibition remained preserved when I_f_ current was inhibited by either ivabradine or Cs^+^ and remained similar to that in control conditions [11] (Figure 10F). 

These results are consistent with the recent report in mouse SANC, which studied effects of selective PDE3 or PDE4 inhibitors on parameters of I_f_ current in the basal state [132]. This study demonstrated that both IBMX and PDE4 inhibitor rolipram caused identical shift of the midpoint activation voltage of I_f_ current by ~15 mV to more positive potentials, while PDE3 inhibitor milrinone had almost no effect even at the high 50 µmol/L concentration [132]. Since milrinone at 50 µmol/L inhibits both PDE3 and PDE4 [91], it is likely that I_f_ current is not regulated by dual PDE3 + PDE4 activation. Indeed, it was concluded that under basal conditions PDE3 and PDE4 create distinct cAMP signaling compartments that differentially regulate access of cAMP to f-channels: PDE4 family alone restricts access of cAMP while PDE3 works in a PKA-dependent manner and remains dormant [132]. The distribution of I_f_ channels within lipid raft domains of SANC [133], which have less permeable and less fluid environment, might produce additional spatial barriers shielding I_f_ channels from cAMP elevation created by PDE3 inhibition. 

## 11. Contribution of LCRs in the Positive Chronotropic Effect of Dual PDE3 + PDE4 Inhibition, Role of Phosphorylation

**Figure 11:**
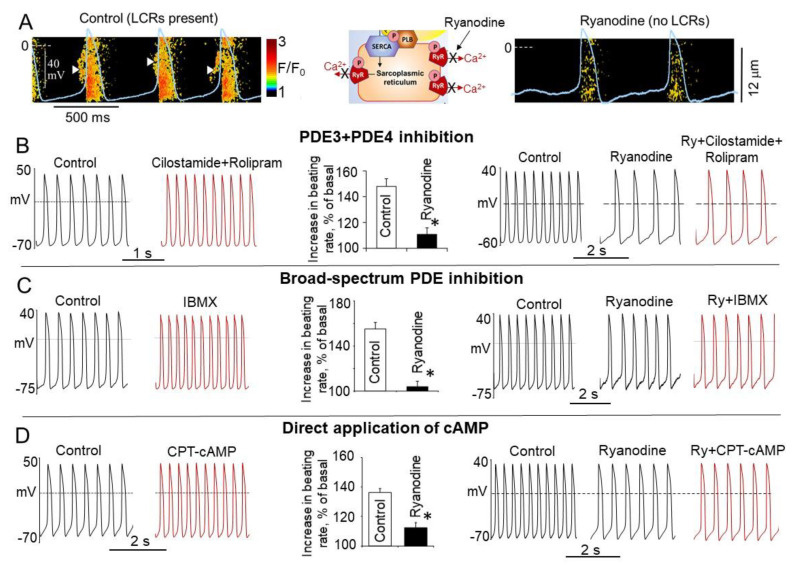
PDE inhibition or direct elevation of (cAMP) in SANC requires intact RyRs function to produce acceleration of the spontaneous beating rate of rabbit SANCs. (**A**) Simultaneous recordings of APs and line-scan images of AP-induced Ca^2+^ transients and LCRs in rabbit SANCs in the absence (left) or presence (right) of ryanodine (3 μmol/L). Suppression of local subsarcolemmal Ca^2+^ releases (LCRs) by ryanodine prevents an increase in the spontaneous SANC firing rate by: (**B**) dual PDE3 + PDE4 inhibition (0.3 μmol/L cilostamide + 2 μmol/L rolipram) (control (n = 4 SANC); ryanodine (n = 7 SANC) *t*-test, * *p* < 0.05, from [11]) or (**C**) by broad-spectrum PDE inhibitor 100 μmol/L IBMX (control (n = 10 SANC); ryanodine (n = 4 SANC) *t*-test, * *p* < 0.05) or (**D**) by direct increase of intracellular cAMP by 300 μmol/L CPT-cAMP (control (n = 4 SANC); ryanodine (n = 7 SANC) *t*-test, * *p* < 0.05; modified from [40]), see text for details.

The strong correlation between protein phosphorylation (reflected in the phosphorylation of PLB), LCRs and the spontaneous SANC beating rate (Figure 8 and Figure 9) indicates that PKA-dependent effects on SR Ca^2+^ cycling, i.e., LCR generation, are required for the PKA-dependent regulation of the SANC beating rate. Therefore, disabling normal SR function while leaving the cAMP/PKA signaling cascade intact ought to interfere with the ability of cAMP/PKA signaling to increase the beating rate. This hypothesis has been verified using ryanodine that locks RyRs in a sub-conductance open state, depleting the SR Ca^2+^ content and eventually eliminating LCRs (Figure 11A, right). 

In the presence of ryanodine, the ability of dual PDE3 + PDE4 inhibition to increase the spontaneous SANC beating rate was reduced from ~50% to only ~10%, indicating a critical role of LCRs for dual (PDE3 + PDE4)-dependent regulation of cardiac pacemaker function (Figure 11B). The similar effect has been also observed with the broad-spectrum PDE inhibitor IBMX (Figure 11C), indicating critical role of RyR Ca^2+^ release, i.e., LCRs, irrespective of whether PDE inhibition is executed at the local level, as likely with dual PDE3 + PDE4 inhibition, or at the global level, as with IBMX. Therefore, increased SERCA2 pumping and RyR Ca^2+^ release within “Ca^2+^ clock” of the coupled-clock system boosts LCR signal mass at earlier times during DD and provides an essential link that connects Ca^2+^ influx via “membrane clock” proteins in response to dual PDE3 + PDE4 inhibition to increase the spontaneous SANC beating rate [11].

**Figure 12:**
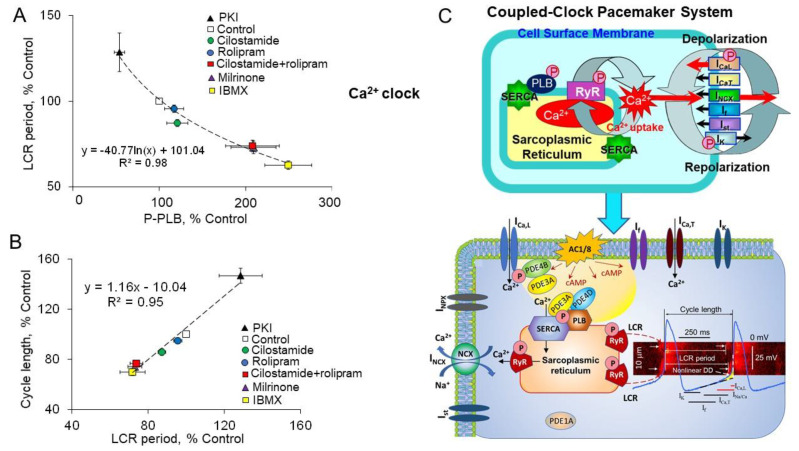
Regulation of the LCR period and spontaneous SANC cycle length through phosphorylation-dependent mechanism. (**A**) The relative effects of a PKA inhibitor PKI (5 µmol/L, n = 4 SANC), PDE3 (0.3 µmol/L cilostamide, n = 4 SANC), or PDE4 (2 µmol/L rolipram, n = 4 SANC) inhibitors alone or dual PDE3 + PDE4 inhibition (n = 7 SANC), IBMX (100 µmol/L, n = 5 SANC) or PDE3 inhibitor milrinone (50 µmol/L, n = 6 SANC) to alter the LCR period are linked to their effects to alter PLB phosphorylation at PKA-dependent Ser^16^ site. (**B**) The relative effects of PKI and diverse PDE inhibitors to alter the spontaneous cycle length are linked to their effects on the LCR period. Note that the effects of dual PDE3 + PDE4 inhibition to alter PLB phosphorylation or the LCR period or cycle length are identical to effects of high (50 µmol/L) milrinone concentration (**A**,**B**, modified from [11,40,41]). (**C**) Top: schematic presentation of the “coupled-clock” pacemaker system; bottom: fine regulation of the “coupled-clock” system by high basal cAMP level, created by constitutive Ca^2+^-activated ACs (AC1 and AC8), which is under tight control of constitutive dual PDE3 and PDE4 activation that works in concert to decrease cAMP and cAMP-mediated PKA-dependent phosphorylation at key locations, i.e., L-type Ca^2+^ channels and SERCA-PLB complex (see text for details).

To assess whether cAMP-dependent modulation of spontaneous SANC firing could operate in the absence of RyR Ca^2+^ release, effects of membrane permeable cAMP analogue (CPT-cAMP) were studied in the absence or presence of ryanodine. The ability of CPT-cAMP to increase the SANC beating rate in the presence of ryanodine was markedly reduced (Figure 12C) [40]. Specifically, under basal conditions CPT-cAMP increased the spontaneous beating rate of rabbit SANC by ~36%, an effect comparable to that of the saturating concentration of the β-AR agonist ISO [40], while in the presence of Ry, the increase of the spontaneous SANC beating rate reached only ~13%, confirming the critical role of RyR Ca^2+^ release in the modulation of spontaneous SANC firing by cAMP/PKA signaling [40].

These results presented in Figure 11D, however, are different from the study by DiFrancesco’s group that showed similar acceleration of the spontaneous SANC beating rate ~17% either in the absence or presence of ryanodine [134]. The differences between the two studies and the small effect of CPT-cAMP on the spontaneous SANC beating rate in the study by DiFrancesco’s group could be explained by the short time (~1 min) of the CPT-cAMP application, which was not sufficient to reach the steady state effect of the rate increase [134]. The steady state level and full-functional effect (~36% acceleration of the spontaneous SANC beating rate) was achieved after ~2-min of CPT-cAMP application [40].

Cardiac pacemaker cells represent a unique system with constitutive AC activity, increased level of cAMP, and PKA-dependent phosphorylation in the basal state, conditions that could be achieved in ventricular myocytes only during β-AR stimulation. The high basal cAMP in cardiac pacemaker cells is not directly linked to normal spontaneous beating of SANC, but is fulfilled through modulation of PKA- and likely CaMKII-dependent protein phosphorylations and normal SR Ca^2+^ function that it supports. Indeed, graded decrease in basal PKA-dependent PLB phosphorylation caused by graded increase in concentrations of PKA-inhibitor peptide PKI are highly correlated with decrease in the spontaneous SANC beating rate [40]. Figure 12A illustrates the dependence of the LCR period on PKA-dependent protein phosphorylation, indexed by phosphorylation of PLB at Ser^16^ site, across a broad range of different interventions. Changes in the phosphorylation status of proteins within the coupled-clock system produced by PKA inhibitor peptide PKI, inhibition of PDE3 or PDE4 alone or dual PDE3 + PDE4 inhibition or IBMX result in reverse changes in LCR periods (Figure 12A), which are paralleled by changes in spontaneous SANC cycle lengths [11,40,41] (Figure 12B).

Moreover, the effects of PDE3 inhibitor milrinone at high 50 µmol/L concentration closely resemble effects of concurrent PDE3 + PDE4 inhibition: both create similar increases in PLB phosphorylation and similar shortenings of the LCR period (Figure 12A), paralleled by similar reductions of the spontaneous SANC cycle lengths (Figure 12B). These results agree with the previous report that 50 µmol/L milrinone inhibits 99% of PDE3A and 75% PDE4D, demonstrating greater effect of milrinone when compared to other PDE3 inhibitors [91]. 

Figure 12C (top) shows a cartoon of the coupled-clock pacemaker system, including the “membrane clock” and “Ca^2+^ clock” with several important phosphorylation sites. There is a complex crosstalk between the two clocks via the signaling pathways that can modulate each other via multiple mechanisms critically dependent on cAMP and the PKA-dependent protein phosphorylation (see chapters above). Figure 12C (bottom) depicts the fine structure of the “coupled-clock” pacemaker system, including our acquired knowledge of how dual basal PDE3 and PDE4 activation, working in a synergistic manner, restricts access of cAMP to the essential locations and, therefore, restrains phosphorylation at SERCA-PLB complex, L-type Ca^2+^ channels, and likely other targets. Both PDE3 and PDE4 are colocalized with L-type Ca^2+^ channels (PDE3A and PDE4B, Figure 6) and SERCA-PLB complex (PDE3A and PDE4D, Figure 7), and neighboring distribution of PDE3 and PDE4 in SANC should support their mutual interactions. Proteins of the “membrane clock” and “Ca^2+^ clock” within the “coupled-clock” pacemaker system interact with each other through mutual entrainment constantly fine-tuning and adjusting to the behavior of the other clock. Basal dual PDE3 + PDE4 activation, by keeping the “coupled-clock” pacemaker system under control, prevents overheating of AC activated cAMP-PKA-dependent signaling through decrease in cAMP and PKA-dependent protein phosphorylation. Specifically, dual PDE3 + PDE4 activation reduces I_Ca,L_, leading to decrease in the SR Ca^2+^ load and local RyR Ca^2+^ release beneath sarcolemma; it also prolongs the SR Ca^2+^ replenishment time, shifting appearance of LCRs to later times. As a result, LCR-activated I_NCX_ is reduced and appeared later during DD restraining positive effects of LCRs to increase the DD rate and spontaneous SANC firing. In this context, LCRs could be envisioned as integrators of multiple PDE-regulated Ca^2+^-AC-cAMP-PKA-dependent functions to ensure the stability of normal spontaneous beating and to safeguard coordinated responses when a change in the beating rate is required. 

## 12. Concluding Remarks

Resting heart rate in humans is an established risk factor for cardiovascular disease [135]. A report based on the Cardiovascular Health Study concluded that elevation in the resting heart rate over six years is associated with an increased risk of cardiovascular disease and mortality [136]. In patients with atrial fibrillation, total PDE activity is ~25% lower and PDE4 activity is ~48% lower compared with those in sinus rhythm, suggesting that loss of PDE activity might contribute to the risk of atrial fibrillation [99].

Similar to other species, the resting rate of the human heart is generated by the SA node, i.e., automaticity of human SANC, which is driven by the coupled-clock pacemaker system regulated by Ca^2+^-cAMP-PKA signaling [137]. Cardiac pacemaker cells have a high basal level of cAMP and PKA-dependent phosphorylation, which is kept in check by high basal PDE activity [2,41]. This review has summarized results of many studies, which showed that the basal spontaneous beating rate of SA nodes of various species is regulated by dual synergistic PDE3 + PDE4 activation (Figure 4D), strongly suggesting that the same mechanism could also regulate the resting rate of the human heart. Though information regarding major PDE subtypes in the human SA node is still missing, PDE3 and PDE4 represent major PDE activities in the SA nodes across multiple species [91,93,94,95], signifying that the same PDE subtypes could play a major role in the human SA node. Though this hypothesis requires further verification, resemblance between regulation of the spontaneous SA node beating rate by dual PDE3 + PDE4 activation across different species (Figure 4D), similarities in the distribution of PDE3 and PDE4 in human cardiac myocytes and SANC (Figure 7), as well as close resemblance of the regulation of I_Ca,L_ by dual PDE3 + PDE4 activation in SANC and human atrial myocytes (Figure 5) [98] provide some support for this idea. In many respects, SANC are close to atrial myocytes [56], and in human atrial myocytes, major PDE activities are represented by PDE3 and PDE4, and dual PDE3 + PDE4 activation regulates the basal cAMP level in a synergistic manner [99]. Future studies that employ human SANC and advanced methods of local cAMP measurements are required to verify whether synergism of dual PDE3 + PDE4 activation regulates basal spontaneous beating of the human heart. It is essential to understand not only general mechanisms of PDE3 and PDE4 interactions and subsequent functional effects, but also interactions of specific PDE3 and PDE4 subtypes. Unfortunately, no pharmacological tools currently exist to clarify functional contributions of specific PDE3 and PDE4 isoforms.
